# Embryo-Induced Changes in the Protein Profile of Bovine Oviductal Extracellular Vesicles

**DOI:** 10.1016/j.mcpro.2025.100935

**Published:** 2025-02-28

**Authors:** Rosane Mazzarella, José María Sánchez, Beatriz Fernandez-Fuertes, Sandra Guisado Egido, Michael McDonald, Alberto Álvarez-Barrientos, Esperanza González, Juan Manuel Falcón-Pérez, Mikel Azkargorta, Félix Elortza, Maria Encina González, Pat Lonergan, Dimitrios Rizos

**Affiliations:** 1 Department of Animal Reproduction, INIA-CSIC, Madrid, Spain; 2 School of Agriculture and Food Science, University College Dublin, Belfield, Dublin, Ireland; 3 Servicio de Técnicas Aplicadas a la Biociencia, Universidad de Extremadura, Badajoz, Spain; 4 Exosomes Laboratory, Center for Cooperative Research in Biosciences (CIC bioGUNE), Basque Research and Technology Alliance (BRTA), Derio, Spain; 5 IKERBASQUE, Basque Foundation for Science, Bilbao, Spain; 6 Centro de Investigación Biomédica en Red en el Área temática de Enfermedades Hepáticas (CIBEReh), Madrid, Spain; 7 Proteomics Platform, Center for Cooperative Research in Biosciences (CIC bioGUNE), Basque Research and Technology Alliance (BRTA), Derio, Spain; 8 Department of Anatomy and Embryology, Veterinary Faculty, Complutense University of Madrid (UCM), Madrid, Spain

**Keywords:** bovine, exracellular vesicles, embryo-maternal interaction, in vivo, in vitro, oviduct, pregnancy

## Abstract

The study of early maternal-embryonic cross-talk remains one of the most challenging topics in reproductive biology. Understanding the physiological mechanisms involved in the interactions between the maternal reproductive tract and the developing embryo is essential for enhancing bovine reproductive efficiency. This complex communication starts within the oviduct, where the modulation of biological processes important for ensuring embryo quality is partially facilitated through extracellular vesicles (EVs). Utilizing a combination of *in vivo* and *in vitro* models this study had three main objectives: 1) to examine the protein cargo of EVs isolated from the oviductal fluid (OF) of cyclic and pregnant heifers to understand their role in maternal-embryonic communication *in vivo*; 2) to characterize the protein profile of EVs in conditioned medium (CM) resulting from the culture of oviductal explants alone (Exp) or in the presence of 8- to 16-cell stage embryos (Exp + Emb); and 3) to compare the protein cargo of EVs from Exp with EVs from cyclic heifers and EVs from Exp + Emb with EVs from pregnant heifers. Proteins were considered “identified” if detected in at least three out of five replicates and considered “exclusive” if detected in at least three out of five replicates within one group but absent in all samples of other groups. We identified 659 and 1476 proteins in the OF-EVs of cyclic and pregnant heifers, respectively. Among these, 644 proteins were identified in OF-EVs from both cyclic and pregnant heifers, and 40 proteins were exclusive to OF-EVs from the pregnant group. Within the 644 proteins identified in both groups, 31 were identified as differently abundant proteins (DAPs). In pregnant heifers, DAPs were mainly related to genome activation, DNA repair, embryonic cell differentiation, migration, and immune tolerance. *In vitro*, we identified 841 proteins in the CM-EVs from Exp alone, 613 from Exp + Emb, and 111 in the CM-EVs from Emb alone. In the qualitative analysis between the three *in vitro* groups, 81 proteins were identified in all groups, 452 were common to Exp and Exp + Emb, 17 were common to Exp and Emb, 5 were common to Exp + Emb and Emb, 4 were unique to Exp, 6 were unique to Exp + Emb, and none were unique to Emb. Proteins identified when there is an interaction between the oviduct and the embryo *in vitro*, corresponding to the Exp + Emb group, were associated with immune tolerance, structural activity, binding, and cytoskeletal regulation. *In vivo* and *in vitro* EVs exhibit distinct qualitative and quantitative protein contents, both when comparing EVs produced in the absence of an embryo (Cyclic and Exp) and those that have undergone embryo-oviduct interaction (Pregnant and Exp + Emb). The observed changes in the protein cargo of EVs due to maternal-embryonic communication *in vivo* and *in vitro* suggest that the interaction between the embryo and the maternal milieu initiates within the oviduct and is potentially facilitated by EVs and their protein contents.

## Highlights

• Characterization of embryo-induced changes in the protein profile of oviductal EVs *in vitro* and *in vivo*.
• Differentially abundant proteins in pregnant heifers are mainly related to genome activation, DNA repair, embryonic cell differentiation, migration, and immune tolerance.
• *In vitro* changes due to embryo-maternal interaction are associated with immune tolerance, structural activity, molecular binding, and cytoskeletal regulation.
• *In vivo* and *in vitro* oviductal EVs exhibit qualitative and quantitative differences in their protein profile.
• The initiation of embryo-maternal dialogue within the oviduct, potentially facilitated by EVs and their protein cargo, contributes to the modulation of biological processes essential for ensuring embryo quality and successful early development.

## Graphical Abstract

**Figure:**
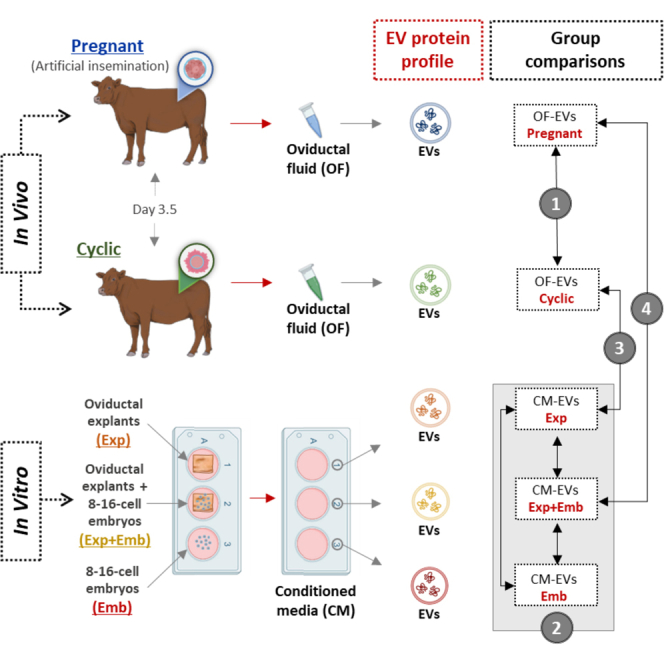

## In brief

This study identified the protein content within oviductal extracellular vesicles as key mediators of early maternal-embryonic communication in the bovine oviduct, revealing pregnancy-specific protein signatures and their potential role in embryo development *in vivo* and *in vitro*.

Proper maternal embryonic cross-talk is critical for the establishment and maintenance of pregnancy. Although successful embryo development up to the hatched blastocyst stage can be achieved *in vitro*, the lack of interaction with the female reproductive tract (oviduct and uterus) leads to inferior embryo quality compared to embryos produced *in vivo* (1). Embryos produced *in vitro* exhibit altered gene expression (2), modified metabolism (3), reduced cryotolerance (4), higher lipid content (5), and diminished pregnancy rates (6) compared to *in vivo* embryos. Hence, it is imperative to elucidate the physiology of the bovine reproductive tract is essential to ultimately improve culture conditions *in vitro*. Specific attention should be given to the oviduct, where fertilization and the initial stages of embryonic development occur.

The oviduct provides the ideal environment for early embryo development, and there is evidence that the embryo interacts with the oviductal microenvironment (7, 8). Following fertilization, early embryo development is sustained by factors produced by the oviductal epithelium and its secretions, the oviductal fluid (OF), providing the embryo with the necessary physiological and biochemical environment for initial development (9). During this period, the first mitotic division occurs, along with metabolic alterations and bovine embryonic genome activation (EGA) around the 8- to 16-cell stage (10). In cattle, the embryo remains in direct contact through its zona pellucida with oviductal epithelial cells and secretions for approximately 3 to 4 days (11).

Extracellular vesicles (EVs) are nanoscale particles comprised of a lipid bilayer secreted by cells into the extracellular environment in response to specific physiological or pathological stimuli (12). Through their bioactive cargo, including proteins (13), lipids (14), mRNAs, and miRNAs (miRNAs) (15), EVs play a crucial role in regulating recipient cells and facilitating cell-to-cell communication. Bovine oviductal EVs have been identified *in vivo* as constituents of the OF (8, 16, 17) and *in vitro* secreted by bovine oviductal epithelial cells (BOECs) (17, 18). The contents of OF-EVs, including proteins, mRNAs, small ncRNAs (19), and miRNAs (20) have been explored across the estrous cycle. Moreover, changes in the miRNA content in cyclic and pregnant cows have been described (8). Also, the protein cargo of oviductal EVs has been analyzed in other species, including cats (21) and pigs (22). Additionally, EVs originating from embryonic sources have been identified in the medium conditioned by bovine embryos *in vitro*, suggesting their potential involvement in maternal cross-talk (23).

Functionally, oviductal EVs from OF are internalized by bovine embryos (24) and improve early embryo development and quality *in vitro*. Lopera-Vásquez *et al*. (18) reported that EVs from BOEC-conditioned medium improved blastocyst quality and induced cryoprotection when supplemented with the embryo *in vitro* culture medium. Additionally, incubating embryos with isthmus-derived EVs improved embryo quality and survival following vitrification (18). Moreover, Almiñana *et al*. (17) demonstrated that OF-EVs improved embryo quality by enhancing their ability to reach the blastocyst stage and hatch. The same group also reported that the uptake of oviductal EVs by *in vitro*-produced embryos is associated with changes in the embryonic transcriptome (25). In a recent study from our group, the sequential supplementation of EVs from OF and uterine fluid (UF) during *in vitro* culture of bovine embryos improved embryo quality by increasing cell number and reducing lipid content in blastocysts (24). These effects were partially attributed to the modulation of gene expression associated with lipid metabolism by the miRNA contained in these EVs (26).

Together, these studies highlight the potential role of EVs in improving embryo quality through embryo-maternal communication, emphasizing the necessity for a more profound understanding of how embryos modulate de cargo of maternal EVs *in vivo*. Additionally, it emphasizes the importance of developing an appropriate *in vitro* model that mimics the physiological environment of the oviduct for studying this complex process of maternal-embryonic cross-talk. Therefore, the present study aimed to 1) analyze the protein content of EVs isolated from OF of pregnant and cyclic heifers to elucidate their role in maternal-embryonic communication *in vivo*; 2) characterize the protein profile of EVs generated from the interaction between embryos and maternal tissue using an oviductal explant model; and 3) compare the protein content between *in vivo* and *in vitro* models.

## Materials and Methods

### Experimental Design

**Fig. 1:**
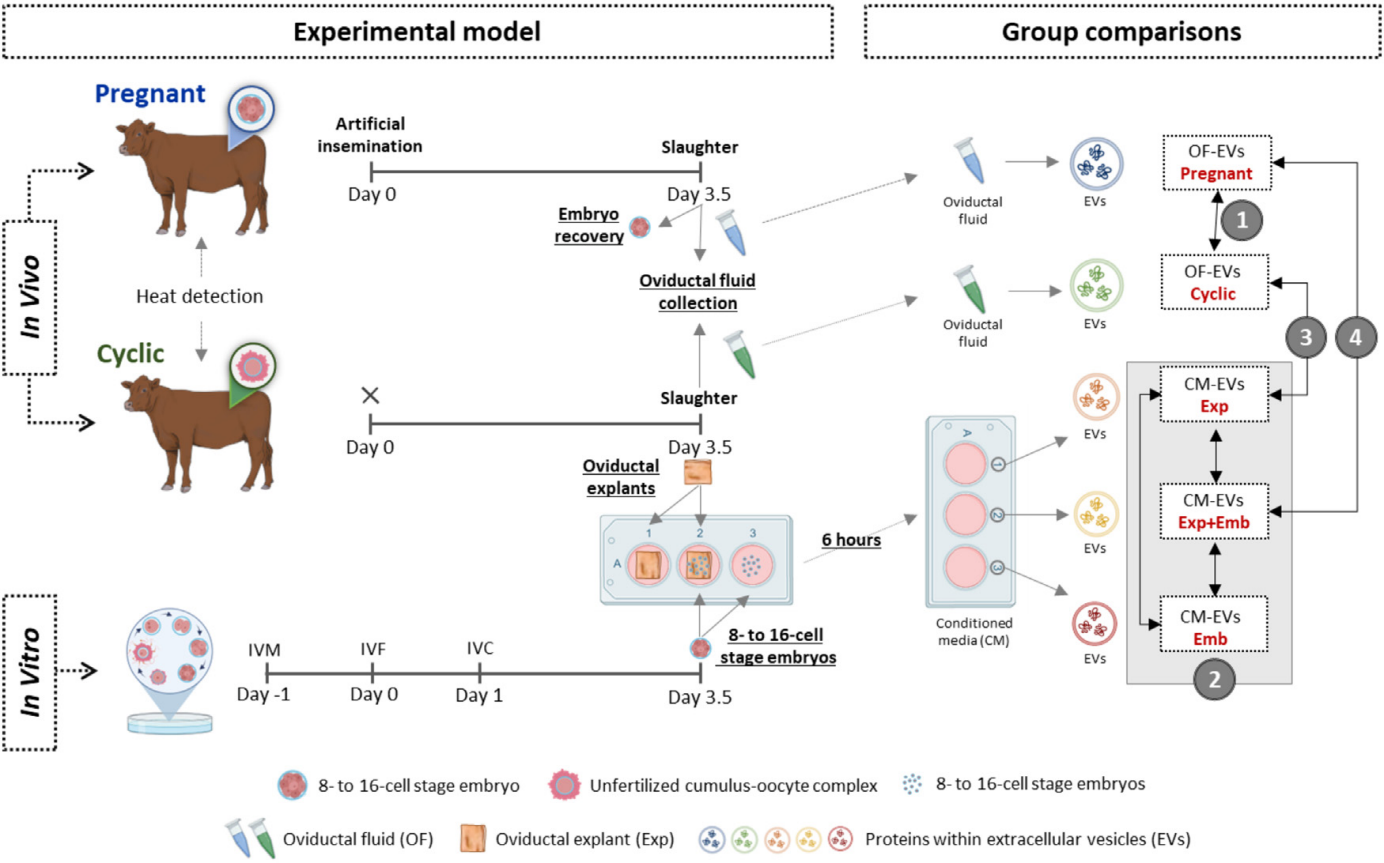
**Experimental Model and Group Comparisons.** Heifers were synchronized, artificially inseminated (Pregnant) or not (Cyclic), and slaughtered 3.5 days after insemination. Oviducts ipsilateral to the corpus luteum were flushed to obtain the oviductal fluid (OF), and the presence of an embryo confirmed pregnancy. For the *in vitro* model, six 0.25 mm^2^ oviductal explants were obtained from each cyclic heifer and cultured individually in 750 μl protein-free synthetic oviduct fluid (SOF): three were cultured alone, and three were co-cultured with 10 *in vitro*-produced 8- to 16-cell stage bovine embryos. Also, a group of 100 *in vitro*-produced 8- to 16-cell stage bovine embryos were cultured alone in 500 μl of SOF. After 6 h, the conditioned media (CM) was collected for extracellular vesicles (EV) isolation. The protein content of EVs from the following four comparisons was analyzed (1): OF-EVs from non-pregnant (Cyclic) heifers compared with pregnant heifers (Pregnant) (2); CM-EVs from oviductal explants cultured alone (Exp) *versus* those co-cultured with 8- to 16-cell stage embryos (Exp + Emb) *versus* the EVs from the CM of 8- to 16-cell stage embryos cultured alone (Emb) (3); OF-EVs from Cyclic heifers compared with EVs from the CM of oviductal explants cultured alone (Exp); and (4) OF-EVs from Pregnant heifers compared with EVs from the CM of oviductal explants co-cultured *in vitro* with 8- to 16-cell stage embryos (Exp + Emb). Created in BioRender (https://BioRender.com/i44h277).

The experimental design is illustrated in Figure 1. We analyzed the protein content of EVs from the following four comparisons:

(1) OF-EVs from non-pregnant (Cyclic) heifers compared with pregnant heifers (Pregnant) to elucidate oviducal EVs role in maternal-embryonic communication *in vivo*.
(2) CM-EVs from oviductal explants cultured alone (Exp) *versus* those co-cultured with 8- to 16-cell stage embryos (Exp + Emb) *versus* EVs from the CM of 8- to 16-cell stage embryos cultured alone (Emb) to elucidate the interaction between embryos and maternal tissue *in vitro*.
(3) OF-EVs from Cyclic heifers compared with EVs from the CM of oviductal explants cultured alone *in vitro* (Exp) to compare the protein content between *in vivo* and *in vitro* models.
(4) OF-EVs from Pregnant heifers with EVs from the CM of oviductal explants co-cultured *in vitro* with 8- to 16-cell stage embryos (Exp + Emb) to compare embryo-maternal communication through EVs *in vivo* and *in vitro*.

### *In vivo* Model

#### Animals

All experimental procedures involving animals were approved by the Animal Research Ethics Committee of University College Dublin and licensed by the Health Products Regulatory Authority, Ireland, in accordance with Statutory Instrument No. 543 of 2012 under Directive 2010/63/EU on the Protection of Animals used for Scientific Purposes.

Crossbred beef heifers (n = 21, predominantly Charolais- and Limousin-cross); 794 ± 52 days old and 578 ± 39 kg (mean ± standard deviation) were synchronized using an 8-day intravaginal P4 device (PRID, 1.55 g P4; Ceva Santé Animale, Libourne, France). On the day of PRID insertion, each heifer received a 2 ml intramuscular injection of an analog of gonadotropin-releasing hormone (GnRH; Ovarelin, Ceva Santé Animale, equivalent to 100 μg gonadorelin). On the day before PRID removal, all heifers received a 5 ml intramuscular injection of an analog of prostaglandin F2 alpha (PGF2α; Enzaprost, Ceva Santé Animale, equivalent to 25 mg dinoprost) to induce luteolysis. Heifers were randomly assigned to be either inseminated (n = 13) at detected estrus to generate pregnancies or not inseminated (n = 8) to generate cyclic controls. Heifers were inseminated twice, approximately 12 and 24 h after the onset of estrus. All heifers were slaughtered at a local abattoir approximately 3.5 days after artificial insemination.

#### OF Collection From Pregnant and Cyclic Heifers

Reproductive tracts were returned to the laboratory on ice within 3 h of slaughter. Oviducts ipsilateral to the corpus luteum (CL) were dissected and separated from the utero-tubal junction. Next, oviducts were flushed with 5 ml of phosphate-buffered saline without Ca^2+^ and Mg^2+^ (PBS^−/−^). The presence of an 8- to 16-cell stage embryo in the oviductal flushing of inseminated heifers was used to confirm pregnancy (n = 5), while the oviductal flushings from non-inseminated heifers contained non-fertilized oocytes and were categorized as cyclic (n = 5). All flushings were centrifuged immediately for 7 min at 300*g* and 4 °C to remove cells. The obtained supernatants were then centrifuged for 30 min at 10,000*g* and 4 °C to remove cellular debris and conserved at −80 °C to be later processed for EV isolation.

### *In vitro* Model

#### Preparation of Oviductal Explants

Oviductal explants were obtained from cyclic heifers and prepared as described by Suarez *et al*. (27) and cultured as described by Mathew *et al*. (28). From the five cyclic heifers, after flushing the oviduct ipsilateral to the CL, the isthmus portion was longitudinally opened, and six 0.25 mm^2^ oviductal pieces of mucosal epithelium and associated underlying stroma were collected from each cyclic heifer. The six explants from the same animal were washed in Hank’s Balanced Salt Solution (HBSS; Gibco, ThermoFisher Scientific) containing 1% antibiotic-antimycotic (ABAM; Gibco, 100X). Subsequently, these explants were individually cultured in a 24-well cell culture plate, with the mucosal epithelium side facing up, in wells containing 750 μl protein-free synthetic oviduct fluid (SOF) and under 5% CO_2_ at 38.5 °C in air with maximum humidity for 2 h before use.

#### Conditioned Medium From Oviductal Explants and 8- to 16-Cell Stage Embryos

Before use, explants obtained as described above were transferred individually to new wells containing 750 μl equilibrated SOF. From the six oviductal explants obtained from each heifer three were cultured in medium alone (Exp), and three were co-cultured with 10 *in vitro*-produced 8- to 16-cell stage bovine embryos each (Exp + Emb). Also, five groups of 100 *in vitro*-produced 8- to 16-cell stage embryos were cultured alone (Emb) in 500 μl of SOF each. All groups were cultured for 6 hours at 5% CO2, 38.5 °C, and maximum humidity. Then, for each cyclic heifer (n = 5), two CM groups were collected (Exp and Exp + Emb) by pooling the CM from their explants cultured alone (Exp) and by pooling the CM from their explants cultured with the embryo (Exp + Emb), forming a total of five pools of Exp and five pools of Exp + Emb. Additionally, CM from the five groups of 100 *in vitro*-produced 8- to 16-cell stage embryos were collected. Finally, CM from all groups was centrifuged for 7 min at 300*g* and 4 °C to remove cells. The obtained supernatant was then centrifuged for 30 min at 10,000*g* and 4 °C to remove cellular debris and conserved at −80 °C to be later processed for EV isolation.

#### *In vitro* Embryo Production

Embryos were produced *in vitro*, as previously described (1). Briefly, bovine immature cumulus-oocyte complexes (COCs) were obtained by aspirating follicles from the ovaries of mature heifers slaughtered at a local abattoir. After selection, COCs were matured during 24 h in groups of 50 per well in 500 μl maturation medium (TCM-199) supplemented with 10% of fetal calf serum (FCS) and 10 ng/ml epidermal growth factor (EGF) at 38.5 °C under an atmosphere of 5% CO_2_ in air with maximum humidity. Matured COCs were fertilized with frozen-thawed sperm from a bull of proven fertility at a concentration of 1 × 10^6^ sperm/ml. Gametes were co-incubated in 500 μl of fertilization medium for 18 to 20 h at 38.5 °C, 5% CO_2_ in air with maximum humidity. Presumptive zygotes were denuded by vortexing and cultured in 500 μl of SOF supplemented with 5% of EV-depleted FCS (dFCS) at 38.5 °C, under 5% CO_2_, 5% O_2,_ and 90% N_2_ with maximum humidity. The dFCS was produced in our laboratory according to the protocol used by Leal *et al*. (2022). Briefly, heat-inactivated FCS (56 °C for 30 min) was ultra-centrifuged at 100,000*g* for 18 h at 4 °C using an Optima-L-100XP Beckman Coulter ultracentrifuge. The supernatant (dFCS) was collected, aliquoted, and stored at − 20 °C for later use. Embryos were recovered 54 h after fertilization at the 8- to 16-cell stage for subsequent use.

### EV Isolation

EVs were isolated from the OF of 5 animals per group (5 Cyclic and 5 Pregnant) and 5 CM per group (5 Exp, 5 Exp + Emb, and 5 Emb). EVs were isolated from the OF and the CM according to the isolation protocol previously reported by our group and based on size-exclusion chromatography (SEC) using PURE-EV (HansaBioMed Life Sciences) (20), an effective method for separating EVs from circulating proteins without altering EV structure or function (29), followed by ultrafiltration using Vivaspin Turbo 15 centrifugal concentrator (Sartorius, 100K MWCO PES). Briefly, after discarding the buffer provided within the SEC column, the column was washed with 30 ml of PBS^−/−^ and then either OF (≈2 ml) or CM (≈2 ml) fluid samples were loaded onto the top of the SEC column. Once the sample was entirely within the column, 11 ml of PBS^−/−^ was loaded, preventing the column from drying out. The EVs were collected in the 2.5 ml fraction after discarding the first 3 ml fraction. Subsequently, the 2.5 ml EV fraction was concentrated by ultrafiltration for 30 min at 2000*g* and 4 °C, resulting in a final volume of 100 μl of concentrated EVs to be used later for EV characterization and proteomic analysis.

### EV Characterization

Following the Minimal Information for Studies of Extracellular Vesicles 2018 guidelines (30), EVs from OF and CM were characterized using flow cytometry (FC), nanoparticle tracking analysis (NTA), and transmission electron microscopy (TEM). FC was performed as previously described by Barranco *et al*. (31), and NTA and TEM as previously described by Leal *et al*. (24). Five *in vivo* and 4 *in vitro* samples per group were utilized for EV characterization.

#### Flow Cytometry

The analyses were conducted following the International Society of Extracellular Vesicles recommendations (MIFlowCyt-EV) (32), utilizing the high-sensitive flow cytometer CytoFLEX S (Beckman Coulter), equipped with violet (405 nm), blue (488 nm), yellow (561 nm), and red (638 nm) lasers. Recombinant EVs expressing GFP (SAE0193, Merck) were used to verify the accuracy of the flow cytometer for EV detection and counting. The optical configuration was optimized to use side scatter (SSC) information from the 405-nm laser (v-SSC). Both the forward scatter (FSC) and v-SSC were set to a logarithmic scale, with the fluorescence channels also adjusted to a logarithmic gain. The analysis was restricted to events with FSC and v-SSC characteristics specific to EVs. Samples were analyzed using the low flow setting (10 μl/min) with a minimum acquisition of 10.10^3^ events per sample. Distilled water (filtered through a 0.1-μm filter) was used as the sheath fluid, and 0.1-μm-filtered PBS^−/−^ was employed to eliminate background noise. Two-minute washing steps with 0.1-μm-filtered distilled water were conducted between EV samples as described in Barranco *et al*. (31). Wach 10 μl EV sample was incubated with CellTrace CFSE (Thermofisher), a non-fluorescent probe that becomes fluorescent upon contact with active esterases present only in functional intact membrane structures, to discriminate intact EVs from membrane fragments. Tetraspanin antibodies anti-CD63-FITC and anti-CD81-APC (REA, Miltenyi Biotec) and anti-CD44-PerCP (Biolegend), with cross-reactivity with bovine species, were used.

#### Nanoparticle Tracking Analysis

The concentration and size distribution of EVs were analyzed using a NanoSight LM-10 system equipped with a CCD video camera and particle-tracking software NTA 3.1 Build 3.1.45 (NanoSight Ltd). Five μl of OF-EVs or CM-EVs solution obtained after SEC were diluted in (1:10) with PBS^−/−^. PBS^−/−^ was used as a negative control. The NTA measurement conditions were detection thresholds 2 to 3, camera level 13, temperature 22 °C, and measurement time 60 s. Three recordings were performed for each sample.

#### Transmission Electron Microscopy

Transmission electron microscopy (TEM) was exclusively employed to confirm the successful isolation of EVs following the established protocol. For that, 5 μl of OF-EVs or CM-EVs solution obtained after SEC were diluted (1:5) with PBS^−/−^ to perform the negative staining of EVs. A carbon-coated collodion 400 mesh nickel grid (Gilder) was floated for 2 min and stained with 2% uranyl acetate (Electron Microscopy Sciences) for 1 min for the negative staining. Grids were visualized in a JEOL JEM 1400 Flash electron microscope (operating at 100 kV). Micrographs were taken with a Gatan OneView digital camera at various magnifications.

### Qualitative and Quantitative Characterization of Proteins

Five samples of EVs from the OF and the CM of each experimental group were used for proteomic analyses. Proteins were considered ‘identified’ when detected in at least three out of five samples in each experimental group and were considered ‘exclusive’ when detected in at least three out of five samples within one group and not detected in the other. Protein quantification was performed using the label-free quantification (LFQ) method integrated into MaxQuant 1.6.17.0 software. Protein abundances were used for both qualitative and quantitative analyses. Quantitative comparisons were performed in pairs: Pregnant *versus* Cyclic, Exp *versus* Emb, Emb *versus* Exp + Emb, Exp *versus* Exp + Emb, Exp *versus* Cyclic and Exp + Emb *versus* Pregnant.

#### In Solution Digestion

Protein was extracted in a sample containing 7 M urea 2 M Thiourea 4% CHAPS and 5 mM DTT, then digested following filter-aided sample preparation protocol described by Wisniewski *et al*. (33) with minor modifications. Trypsin, used to generate peptides through specific cleavage, was added at a trypsin: protein ratio of 1:20, and the mixture was incubated overnight at 37 °C, dried out in an RVC2 25 speed vac concentrator (Christ), and resuspended in 0.1% formic acid. Peptides were desalted and resuspended in 0.1% FA using C18 stage tips (Millipore).

#### Mass Spectrometry Analysis

Samples were analyzed in a timsTOF Pro with PASEF (Bruker Daltonics) coupled online to an Evosep ONE liquid chromatograph (Evosep). A total of 200 ng were directly loaded onto the Evosep ONE and resolved using the 60 samples-per-day protocol. Protein identification and quantification were carried out using the label-free quantification (LFQ) method integrated into MaxQuant 1.6.17.0 software. Searches were carried out against a database consisting of Bos Taurus entries from UniProt Swissprot + TrEMBL (downloaded on April 6, 2022), consisting of 117,111 entries. Carbamidomethylation of cysteines was set as a fixed modification, and oxidation of methionine and N-terminal acetylation of proteins were set as variable modifications. Two missed cleavages were allowed for trypsin digestion. Precursor and fragment tolerances of 20 ppm and 0.05 Da were considered for the searches, respectively. A 1% False Discovery Rate (FDR) was applied at both the PSM (peptide-spectrum match) and protein levels. Only proteins with at least two different Unique + razor peptides were considered for further analysis.

### Statistical and Bioinformatics Analysis

#### EVs Characterization

*In vivo* and *in vitro* data were tested for outliers using the ROUT test and for normality using the Shapiro–Wilk test. The normality was confirmed, and *in vivo* data were analyzed using Student’s *t* test, while *in vitro* data were analyzed using one-way ANOVA followed by the Tukey test. Statistical analyses were performed using GraphPad Prism 10. For all analyses, *p* ≤ 0.05 was considered significant.

#### Proteomics

Protein abundance data were analyzed using Student’s *t* test. For all analyses, a *p* ≤ 0.05 was considered significant for further analyses and discussion. Peak area data were transformed using log2 for graphical representation. Principal Component Analysis (PCA) was generated by Metaboanalyst 6.0 (https://www.metaboanalyst.ca). Venn diagrams were constructed using Venny 2.1 (https://bioinfogp.cnb.csic.es/tools/venny/). Molecular function, biological processes, cellular processes, protein class, and identification of biological pathways of the proteins were evaluated using the PANTHER 18.0 Classification System (https://PANTHERdb.org/) with *Bos taurus* as the selected organism (34). Metascape Membership tool v3.5.20240901 (https://metascape.org) was used to identify significant enrichment (*p* ≤ 0.05) matching the term “embryo development” (35).

## Results

### EVs Characterization

EVs isolated from both *in vivo* and *in vitro* models were characterized for size and concentration by NTA, presence of EV markers (CD63, CD81, and CD44) by flow cytometry, and morphology by TEM.

#### *In vivo* Model

**Fig. 2:**
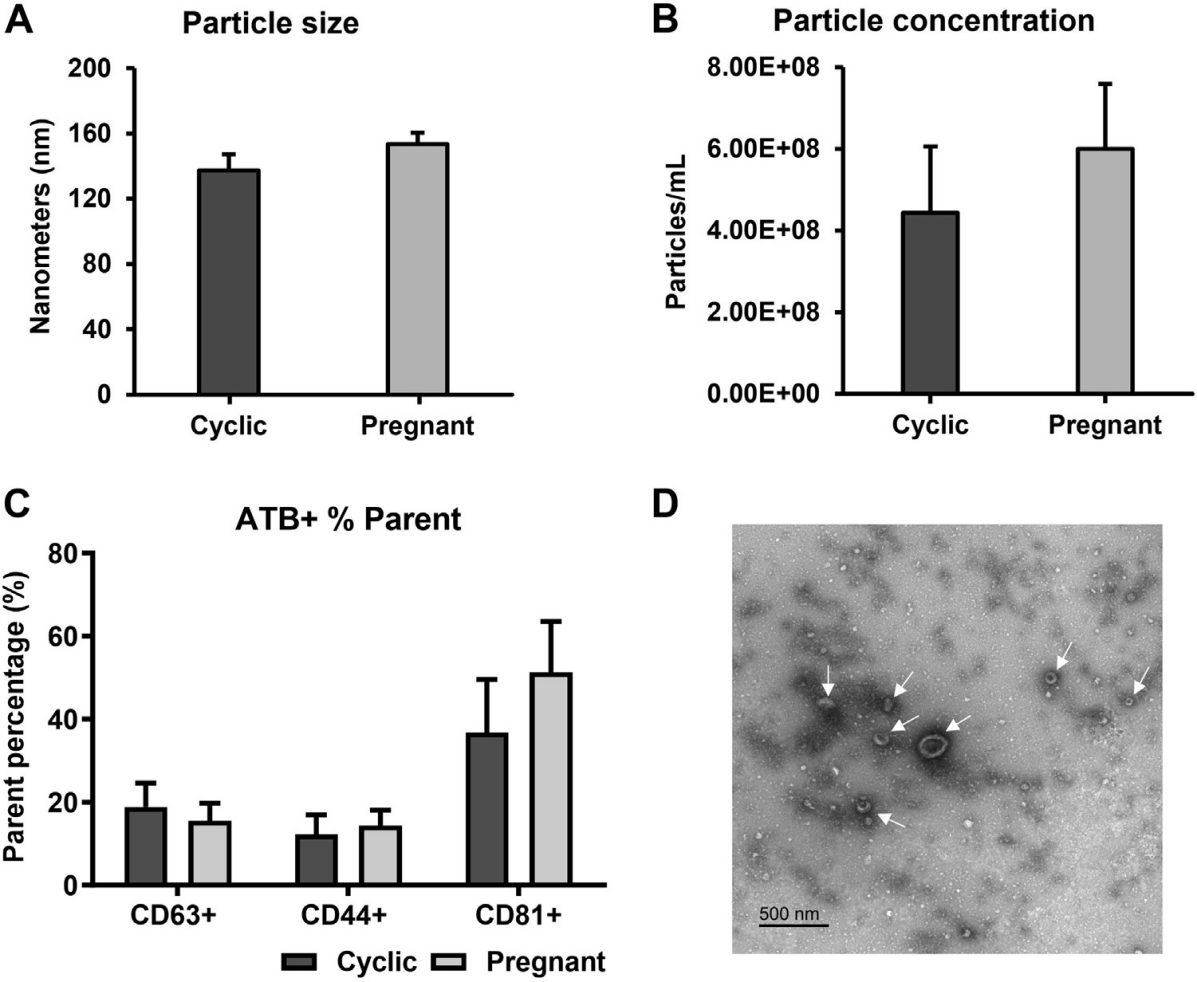
**Characterization of Oviductal Fluid Extracellular Vesicles (OF-EVs).** Nanoparticle tracking analysis showed no difference in particle size (*A*) and concentration (*B*) between the groups Cyclic and Pregnant. *C*, flow cytometry identified the CD63, CD81, and CD44 markers in both groups. *D*, transmission electron microscopy image from OF-EVs showing cup-shaped particles with characteristic sizes resembling EVs. Error bars represent the standard error of the mean (SEM). White arrows indicate EVs.

In the NTA analysis, no differences were identified in particle size (Cyclic: 137 ± 10 nm and Pregnant: 154 ± 7 nm; Fig. 2*A*) and particle concentration (Cyclic: 4.44 × 10^8^ ± 1.63 x 10^8^ particles/ml and Pregnant: 6.00 x 10^8^ ± 1.60 x 10^8^ particles/ml; Fig. 2*B*). The NTA negative control showed zero particles per frame. EV presence was confirmed by flow cytometry with the identification of the CD63, CD81, and CD44 markers in both groups (Fig. 2*C*). TEM images identified the presence of cup-shaped particles with characteristic sizes resembling EVs in the OF (Fig. 2*D*). Therefore, we identified the presence of EVs in the OF of Cyclic and Pregnant heifers and confirmed the efficiency of the isolation protocol.

#### *In vitro* Model

**Fig. 3:**
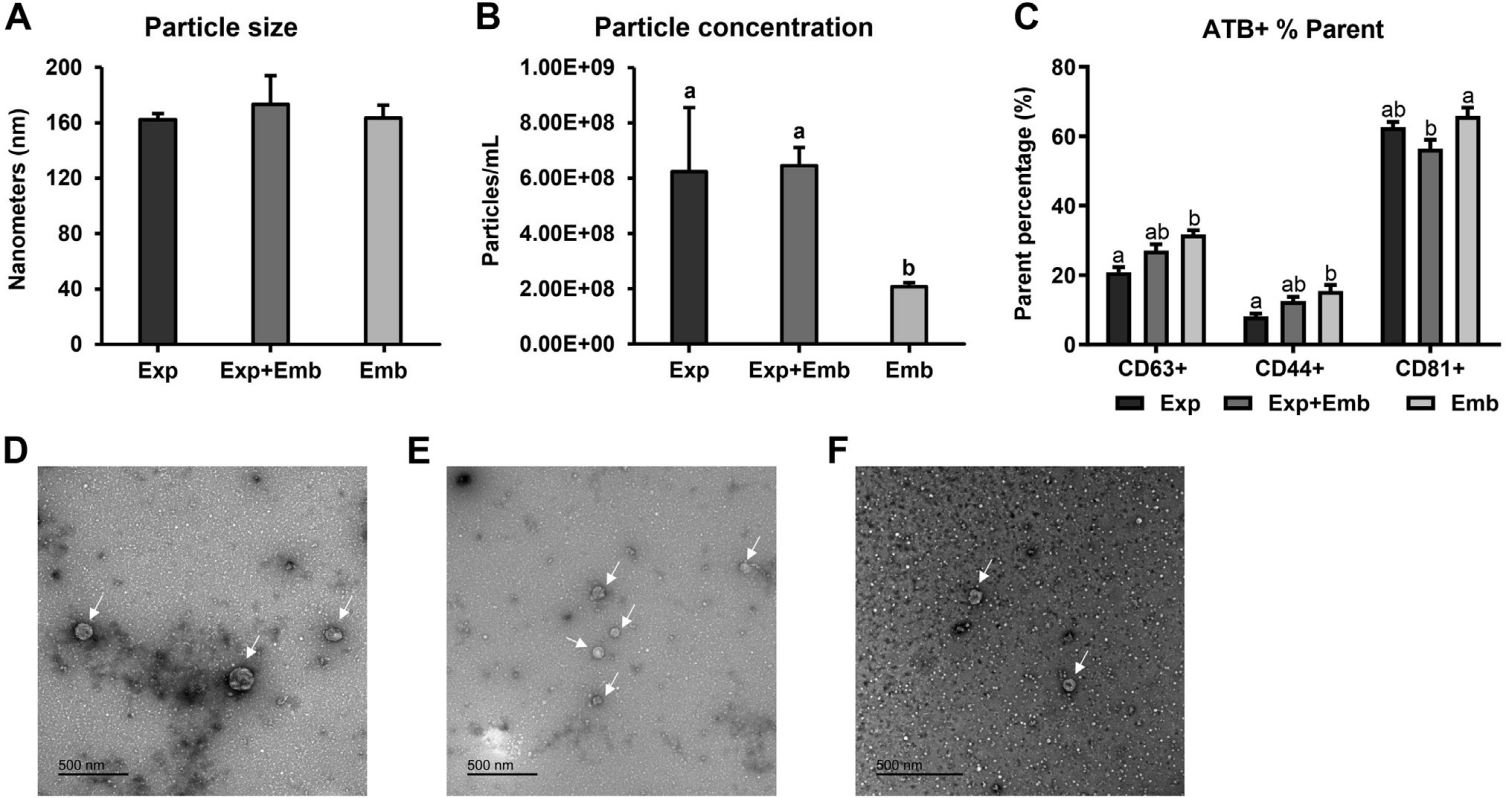
**Characterization of Conditioned Media (CM) Extracellular Vesicles (EVs).** Nanoparticle tracking analysis showing no difference in particle size (*A*) among CM-EVs from oviductal explants cultured alone (Exp), oviductal explants co-cultured with 8- to 16-cell stage embryos (Exp + Emb) and embryos cultured alone (Emb) (*B*). *C*, flow cytometry showing the identification of the CD63, CD81, and CD44 markers in all groups. *D*, transmission electron microscopy image showing cup-shaped particles with characteristic sizes resembling EVs in the CM by Exp (*D*), Exp + Emb (*E*), and Emb (*F*). Error bars represent SEM. *White* arrows indicate EVs. Different letters indicate significative differences (*p* ≤ 0.05).

In the NTA analysis, no differences were identified in particle size among the groups Exp, Exp + Emb, and Emb (Exp: 162 ± 4.4 nm, Exp + Emb: 173 ± 20.6 nm, and Emb: 163 ± 9.39 nm; Fig. 3*A*), while the particle concentration was lower in the CM from Emb (Exp: 6.23 x 10^8^ ± 2.32 x 10^8^ particles/ml, Exp + Emb: 6.45 x 10^8^ ± 6.65 x 10^7^, and Emb: 2.08 x 10^8^ ± 1.41 x 10^7^ particles/ml; Fig. 3*B*). The NTA negative control showed zero particles per frame. EV presence was confirmed by flow cytometry with the identification of the CD63, CD81, and CD44 markers in the three groups (Fig. 3*C*). TEM images identified the presence of cup-shaped particles with characteristic sizes resembling EVs in the CM from Exp, Exp + Emb and Emb (Fig. 3, *D*–*F*). Therefore, we identified the presence of EVs in the CM and confirmed the efficiency of the isolation protocol.

### Qualitative and Quantitative Characterization of Proteins

#### *In vivo* Model

**Fig. 4:**
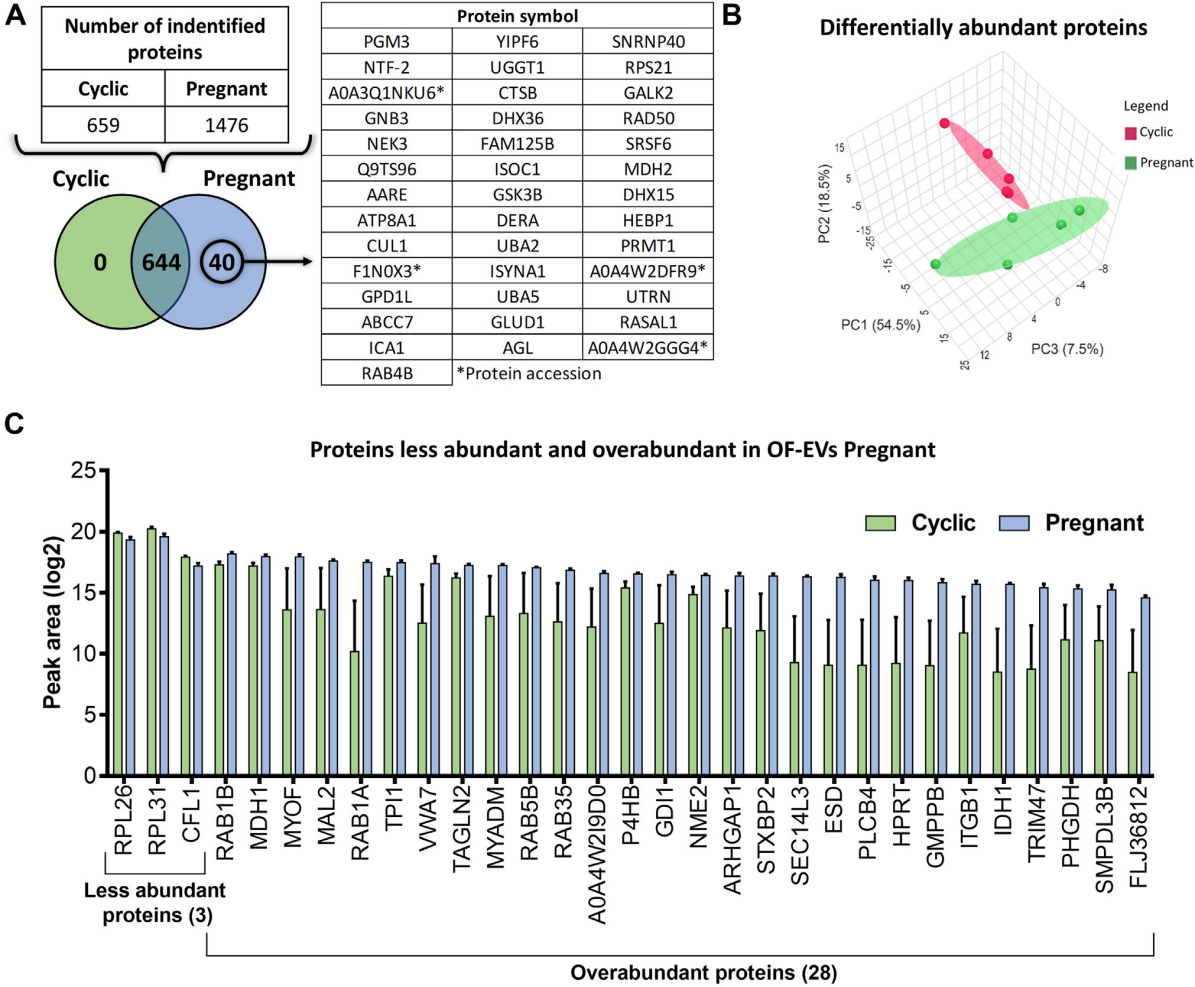
**Protein Profile of Oviductal Fluid Extracellular Vesicles (OF-EVs) from Cyclic and Pregnant heifers.***A*, the table indicates the number of proteins identified in each group, and the Venn diagram represents the 644 proteins common to both and the 40 proteins exclusively detected in Pregnant heifers. *B*, principal Component Analysis of differentially abundant proteins. *C*, three proteins were less abundant, and 28 were more abundant in OF-EVs from Pregnant compared to Cyclic heifers. Proteins were considered ‘identified’ if detected in at least three out of five replicates and considered ‘exclusive’ if detected in at least three out of five replicates within one group but absent in all samples of other groups. Error bars represent SEM. *p* ≤ 0.05 was considered as significant.

We identified 659 proteins in the OF-EVs of Cyclic and 1476 proteins in the OF-EVs from Pregnant heifers (Supplementary file 1, *A* and *B*, respectively). Of these, 644 proteins were commonly identified between the two groups, and 40 were exclusive to OF-EVs from the Pregnant group (Fig. 4*A*). Among the 644 identified in both groups, 31 proteins were differentially abundant (*p* ≤ 0.05; Supplementary file 2): three proteins were less abundant and 28 were more abundant in Pregnant heifers (Fig. 4*C*). Additionally, the PCA plot from the DAPs revealed two distinct clusters of Cyclic and Pregnant heifers (Fig. 4*B*).

Among the top 30 most abundant proteins in Cyclic heifers (Table 1) were proteins such as the oviduct-specific glycoprotein (OVGP1), four annexins (A0A4W2CRW4, ANXA2, ANXA4, and ANXA5), two tubulins (TUBB and TUBB4B), mucin (MUC16) and 10 ribosomal proteins (RPS8, RPL18, RPL7A, RPL7, RPL6, RPS9, RPL31, RPS6, RPL19 and RPS16). In Pregnant heifers (Table 1), among the top 30 most abundant proteins were OVGP1, 6 annexins (A0A4W2CRW3, ANXA2, ANXA4, ANXA5, ANXA11, and LOC113885433), three tubulins (TUBB, TUBB4B, TUBA1D), MUC16, three ribosomal proteins (RPS8, RPL18, and RPL7A). Heat shock protein HSPA8 and RAS protein (RASAL1) were also among the most abundant proteins in Pregnant heifers.

**Table 1:** Top 30 most abundant proteins in the oviductal fluid extracellular vesicles (OF-EVs) from (A) Cyclic and (B) Pregnant heifers

(A) Top 30 most abundant proteins in OF-EVs from cyclic heifers.				
Protein accession	Gene name	Peptides	Razor + unique peptides	Description
A0A4W2FD79	ZNF638	5	5	Zinc finger protein 638
E1BAU6	INPP5E	2	2	Inositol polyphosphate-5-phosphatase E
Q28042	OVGP1	44	44	Oviduct-specific glycoprotein (Fragment)
A0A4W2E8T3	CD109	58	58	CD109 molecule
A0A4W2CRW3	–	27	27	Annexin
A0A4W2HPZ4	RARRES1	16	16	Retinoic acid receptor responder 1
A0A4W2CVQ1	ANXA2	43	43	Annexin
A0A4W2DYQ2	ACTB	35	35	Actin beta
Q3MHM5	TUBB4B	27	7	Tubulin beta-4B chain
A0A4W2HYA4	EZR	54	54	Ezrin
A0A4W2DVZ1	TUBB	28	28	Tubulin beta chain
A0A4W2EXX8	RPS8	14	14	40S ribosomal protein S8
A0A4W2G1A4	KRT76	9	2	Keratin, type II cytoskeletal 2 oral-like
A0A3Q1MHG7	ANXA4	29	29	Annexin
A0A4W2GZL4	IL1RAP	23	23	Interleukin 1 receptor accessory protein
A0A4W2EFE8	RPL18	13	13	Ribosomal protein L18
Q2TBQ5	RPL7A	21	21	60S ribosomal protein L7a
E9LZ03	E9LZ04	32	32	Prominin-1
A0A4W2GAA4	RPL7	28	28	Ribosomal protein L7
A8E4P3	STOM	18	18	STOM protein
F2FB38	MUC16	39	39	Mucin-16
A0A4W2C8R9	CD9	4	4	Tetraspanin
A0A4W2IRD9	ENPP3	28	28	Ectonucleotide pyrophosphatase/phosphodiesterase 3
A0A4W2E7H4	RPL6	27	27	60S ribosomal protein L6
A0A4W2D2Y6	RPS9	25	25	40S ribosomal protein S9
A0A4W2FQP8	RPL31	12	12	60S ribosomal protein L31
A0A4W2EMD8	RPS6	20	20	40S ribosomal protein S6
A0A4W2IRI4	ANXA5	33	33	Annexin
A0A4W2E0U9	RPL19	16	16	Ribosomal protein L19
A0A4W2HV66	RPS16	17	17	Ribosomal protein S16

(B) Top 30 most abundant proteins in OF-EVs from pregnant heifers.				
Protein accession	Gene name	Peptides	Razor + unique peptides	Description
A0A4W2FD79	ZNF638	5	5	Zinc finger protein 638
Q28042	OVGP1	2	2	Oviduct-specific glycoprotein (Fragment)
E1BAU6	INPP5E	44	44	Inositol polyphosphate-5-phosphatase E
A0A4W2IDW3	RASAL1	58	58	RAS protein activator like 1
A0A4W2E8T3	CD109	27	27	CD109 molecule
A0A4W2HPZ4	RARRES1	16	16	Retinoic acid receptor responder 1
A0A4W2CRW3	–	43	43	Annexin
A0A4W2CVQ1	ANXA2	35	35	Annexin
A0A4W2DYQ2	ACTB	27	7	Actin beta
A0A3Q1MHG7	ANXA4	54	54	Annexin
A0A4W2HYA4	EZR	28	28	Ezrin
A0A4W2IRD9	ENPP3	14	14	Ectonucleotide pyrophosphatase/phosphodiesterase 3
A0A4W2DVZ1	TUBB	9	2	Tubulin beta chain
A0A4W2EXX8	RPS8	29	29	40S ribosomal protein S8
Q3MHM5	TUBB4B	23	23	Tubulin beta-4B chain
E9LZ03	–	13	13	Prominin-1
A8E4P3	STOM	21	21	STOM protein
A0A4W2G1A4	KRT76	32	32	Keratin, type II cytoskeletal 2 oral-like
A0A4W2GZL4	IL1RAP	28	28	Interleukin 1 receptor accessory protein
A0A4W2IRI4	ANXA5	18	18	Annexin
A0A4W2EFE8	RPL18	39	39	Ribosomal protein L18
A0A4W2C8R9	CD9	4	4	Tetraspanin
F1MUN7	ANXA11	28	28	Annexin
F2FB38	MUC16	27	27	Mucin-16
Q2TBQ5	RPL7A	25	25	60S ribosomal protein L7a
A0A452DJ66	TUBA1D	12	12	Tubulin alpha chain
A0A4W2ISK3	S100A10	20	20	Calpactin I light chain
P04896	GNAS	33	33	Guanine nucleotide-binding protein G(s) subunit alpha
A0A4W2FJT1	HSPA8	16	16	Heat shock protein family A (Hsp70) member 8
A0A4W2CZY6	LOC113885433	17	17	Annexin

**Fig. 5:**
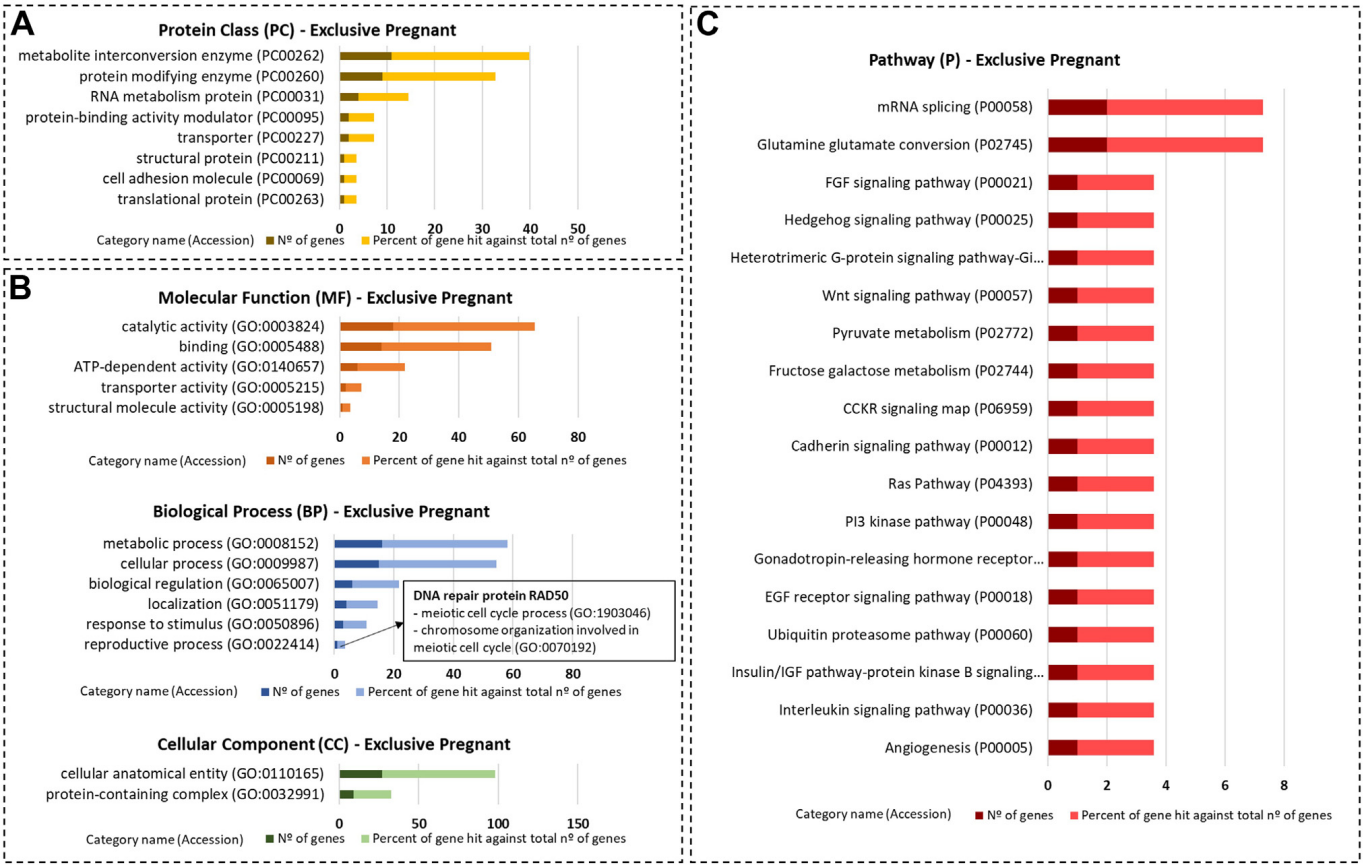
**Functional enrichment of the proteins exclusive to oviductal fluid extracellular vesicles of pregnant heifers.***A*, protein class, (*B*) gene ontology, and (*C*) pathways identified using the PANTHER 18.0 Classification System (https://pantherdb.org/). Darker bars indicate the number of genes associated with each category name, while lighter bars represent the percentage of these genes relative to the total number of genes in that category.

Functional enrichment using the PANTHER database indicated that the identified proteins are involved in a variety of GO biological processes and pathways. Most of the 40 proteins exclusive to OF-EVs of Pregnant heifers belong to the protein class of enzymes related to protein modification and RNA metabolism (Fig. 5*A*). These proteins are related to biological processes such as the reproductive process (DNA repair protein RAD50, Fig. 5*B*) and pathways related to embryo development such as Wnt, Ras, and PI3K-AKT (Fig. 5*C*). Additionally, a search of the literature relating to the proteins among the 40 exclusives to OF-EVs of Pregnant heifers focusing on their known functions in reproductive biology (Table 2) highlighted their association with EGA, DNA repair, the cell cycle, and the Wnt biological pathway.

**Table 2:** Proteins with known functions in reproductive biology among the 40 proteins exclusive to oviductal fluid extracellular vesicles of pregnant heifers

Protein accession	Gene name	Protein description	General function	Overview in reproductive biology	Species	References
A0A4W2DBI2	HEBP1	Heme binding protein 1	Cellular metabolism	Successful embryo development and related to protein synthesis and cell differentiation.	Bovine	(117)
Q32KP9	NUTF2	Nuclear transport factor 2	Structural activity	Unique to UF from Day 16 pregnant heifers and likely originated from the conceptus	Bovine	(118)
A0A4W2EC36	UBA2	SUMO-activating enzyme subunit 2	Protein modification	EGA transition and response to DNA damage in embryos. Adding UBA2 *in vitro* enhanced oocyte maturation and embryo development.	Porcine	(65, 66)
Q32PB8	RPS21	40S ribosomal protein S21	Ribosomal protein	Predictive for blastocyst developmental competency the developmental capacity in ICM and TE cells	Bovine	(119, 120)
A0A4W2HTZ2	RAD50	RAD50 double-strand break repair protein	DNA repair	Telomere protection, DSB recognition, and the activation of cell cycle checkpoints in early embryonic development. Highly activated at EGA, with higher expression in early cleaving than in late cleaving embryos.	Bovine Human Murine Porcine	(61, 62, 63, 121)
A0A4W2H715	CTSB	Cathepsin B	Turnover of proteins	Embryo development, implantation, and placentation. Potential marker for pregnancy detection in the blood cells.	Bovine Equine Murine Porcine	(122, 123, 124, 125)
F6RFE5	PRMT1	Protein arginine methyltransferase 1	Epigenetic transcriptional activation	Transcription activation before EGA. DNA damage check point control, genome integrity, and cell proliferation.	Bovine Murine	(58, 126)
A5D7D9	DHX15	RNA helicase	Pre-mRNA processing factor	EGA at the 8-cell stage and involved in RNA metabolism, such as transcription initiation, ribosome biogenesis, and pre-mRNA splicing.	Bovine Equine	(10, 56)
A0A4W2HFY1	CUL1	Cullin 1	Protein modification	Oocyte maturation, preimplantation embryo development, and EGA.	Bovine Caprine Murine	(55, 127, 128)
A0A4W2FG68	MDH2	Malate dehydrogenase	Energy metabolism	Metabolic process during morula and blastocyst formation. Molecular markers of developmental competence in embryos.	Bovine Ovine	(108, 129)
A0A4W2EPP2	GLUD1	Glutamate dehydrogenase NAD(P) (+)	Energy metabolism	Required for metabolic fine-tuning of the TCA-cycle in the developing embryos. Increased in UF of pregnant versus open heifers.	Bovine Murine	(130, 131)

DSB, doble stand brake; EGA, embryonic genome activation; ICM, inner cell mass; TCA, tricarboxylic acid; TF, trophectoderm; UF, uterine fluid.

**Fig. 6:**
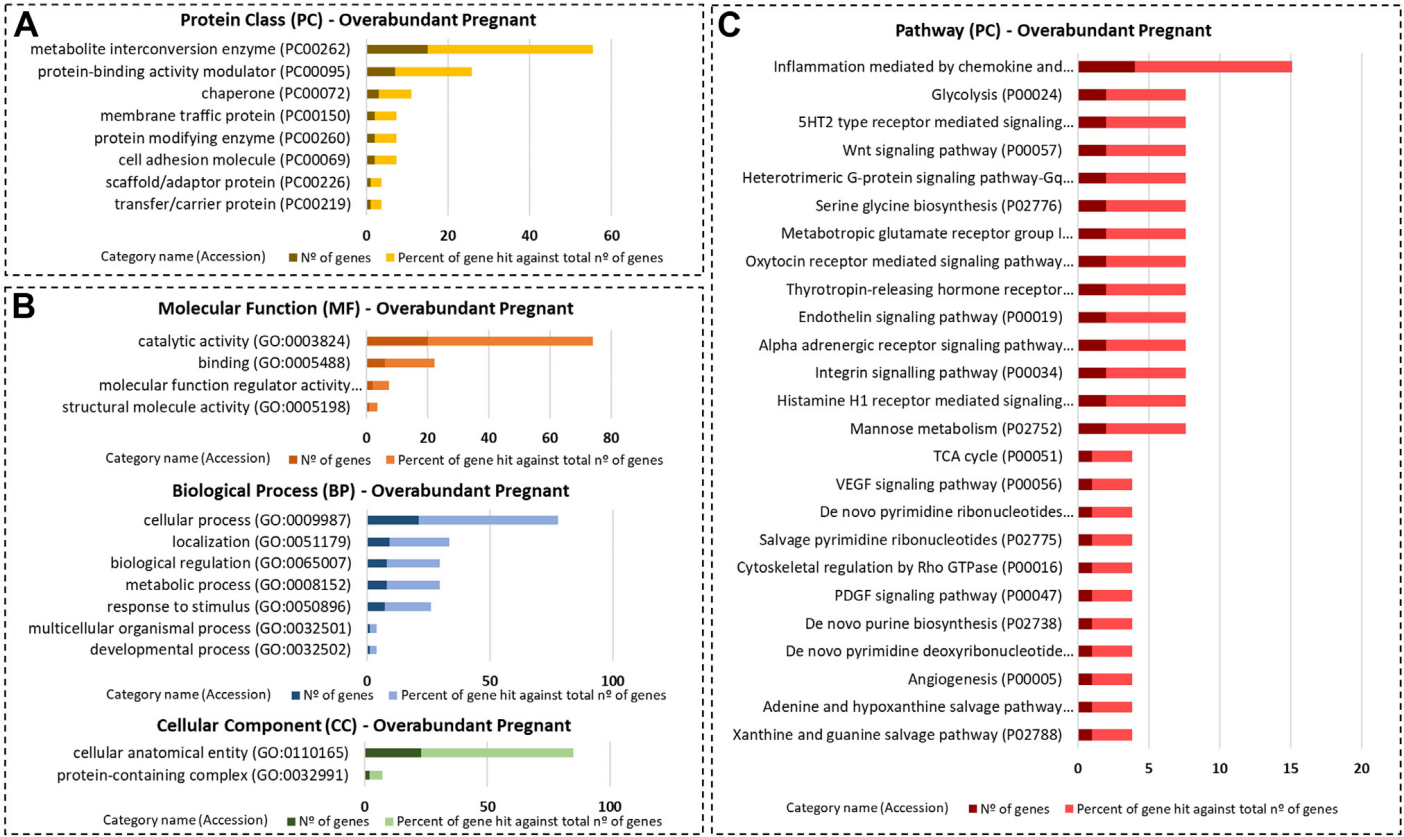
**Functional enrichment of the proteins overabundant abundant in oviductal fluid extracellular vesicles of pregnant compared with cyclic heifers.***A*, protein class, (*B*) gene ontology, and (*C*) pathways identified using the PANTHER 18.0 Classification System (https://pantherdb.org/). Darker bars indicate the number of genes associated with each category name, while lighter bars represent the percentage of these genes relative to the total number of genes in that category.

Most of the 28 proteins overabundant in OF-EVs of Pregnant compared with Cyclic heifers were enzymes and proteins with protein binding activity (Fig. 6*A*). These proteins were also related to cellular, metabolic, and developmental processes (Fig. 6*B*), as well as pathways such as Wnt and glycolysis (Fig. 6*C*). Additionally, among the 28 proteins overabundant in OF-EVs of Pregnant heifers with known functions in reproductive biology were proteins related to pregnancy and proliferation of embryonic cells (Table 3). The three proteins less abundant in OF-EVs of Pregnant compared with Cyclic heifers are structural proteins (Supplemental Fig. S1*A*) related to cellular and metabolic processes (Supplemental Fig. S1*B*) and cytoskeletal regulation (Supplemental Fig. S1*C*). None of these three proteins have been reported to have functions in reproductive biology.

**Table 3:** Proteins with known functions in reproductive biology among the 28 more abundant proteins in oviductal fluid extracellular vesicles of Pregnant heifers

Protein accession	Gene name	Protein description	General function	Reproductive function	Species	References
A0A452DIW4	MDH1	Malate dehydrogenase	Catalytic activity	Oocyte maturation and embryo development. Upregulated in the UF of pregnant versus cyclic mares.	Porcine Equine Murine	(70, 132, 133)
P21856	GDI1	Rab GDP dissociation inhibitor alpha	Protein transport	Unique to UF from pregnant heifers and likely originated from the conceptus.	Bovine	(68)
Q5E956	TPI1	Triosephosphate isomerase	Carbohydrate metabolism	Biomarkers for competence at the bovine blastocyst stage. One of the major cellular proteins of bovine trophectoderm cell lines. Increased in the OF of pregnant mares.	Bovine Equine	(46, 72, 134)
A6H7J6	P4HB	Protein disulfide-isomerase	Protein modification	Unique to UF from pregnant heifers and likely originated from the conceptus.	Bovine	(68, 130)
A0A4W2DF00	TAGLN2	Transgelin	Protein binding	Oocyte maturation. Trophoblast migration, invasion, and fusion. Regulate uterine smooth muscle throughout pregnancy. Identified as interacting with embryos at the morula stage.	Bovine Murine Human	(43, 47, 135, 136)
A0A4W2DW55	NME2	Nucleoside diphosphate kinase	Metabolic enzymes	Overexpressed in bovine OF ipsilateral to the pre-ovulatory follicle.	Bovine	(137)
A0A4W2E583	ITGB1	Integrin beta	Structural activity	Endometrium-embryo communication and embryo attachment.	Bovine Human Ovine Porcine	(138, 139, 140, 141, 142)
F1MHC2	STXBP2	Syntaxin binding protein 2	Vesicle-mediated transport	Identified in bovine oviduct EVs from *in vivo* and *in vitro* origin associated with reproductive roles.	Bovine	(17)
A0A4W2HZM5	ARHGAP1	Rho GTPase activating protein 1	Endosomal transport	Increased on the endometrium of pregnant heifers. Marker for trophoblast differentiation.	Bovine	(143, 144)
A0A4W2HA65	IDH1	Isocitrate dehydrogenase [NADP]	Cellular metabolism	Increased on the endometrium of pregnant heifers. Response to oxidative stress and cellular metabolism to drive rapid proliferation of embryonic cells during the elongation process.	Bovine Equine	(145, 146)

EVs, extracellular vesicles; OF, oviductal fluid; UF, uterine fluid.

#### *In vitro* Model

**Fig. 7:**
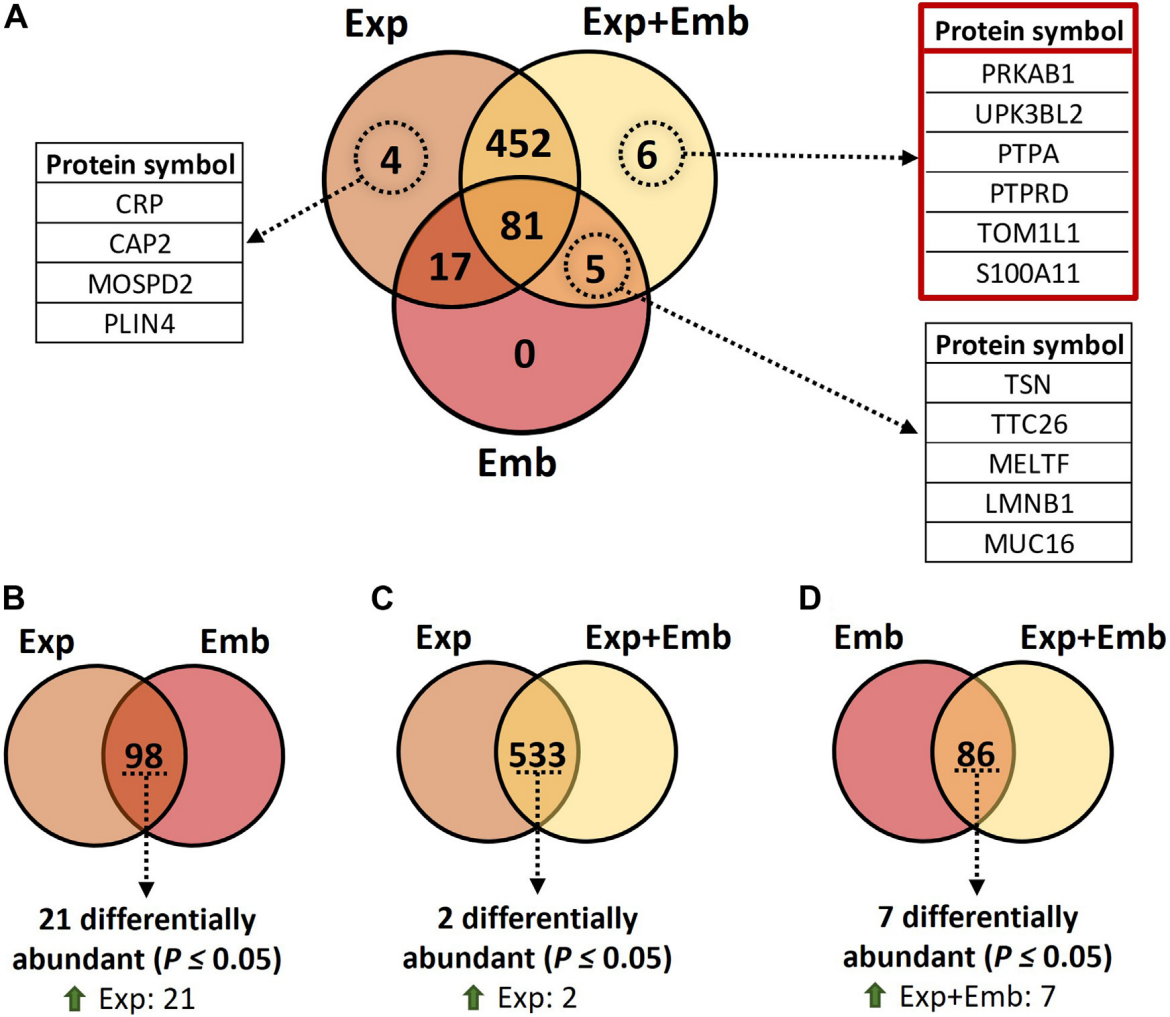
**The protein profile of extracellular vesicles from conditioned media following the culture of oviductal explants in the absence (Exp) or presence (Exp + Emb) of 8- to 16-cell stage embryos, or from embryos cultured alone (Emb).***A*, Venn diagram represents the number of proteins associated with CM-EVs from Exp, Exp + Emb, and Emb. *Red* boxes indicate the list of the six proteins identified as only present when there is an interaction between the oviduct and the embryo *in vitro*. *B*, Venn diagrams representing the number of proteins in common among Exp *versus* Emb, (*C*) Exp *versus* Exp + Emb, and (*D*) Emb *versus* Exp + Emb, as well as the number of differentially abundant proteins amongst the common proteins in each comparison. Proteins were considered ‘identified’ if detected in at least three out of five replicates and considered “exclusive” if detected in at least three out of five replicates within one group but absent in all samples of other groups.

We identified 841 proteins in the CM-EVs from Exp, 613 from Exp + Emb, and 111 from Emb (Supplementary file 3, *A*–*C*, respectively). Of these, 81 proteins were commonly identified among the three groups, 533 were common to Exp and Exp + Emb, 86 were common to Exp and Emb. Four proteins were unique to Exp (CRP, CAP2, MOSPD2, and PLIN4), and none were unique to Emb (Fig. 7*A*). Additionally, six unique proteins (PRKAB1, UPK3BL2, PTPA, PTPRD, TOM1L1, and S100A11) were identified as being exclusively present when there is an interaction between the oviduct and the embryo *in vitro*, in the group Exp + Emb (Fig. 7*A*, red boxes).

Furthermore, the comparisons between Exp and Emb, Exp and Exp + Emb, and Emb and Exp + Emb highlighted distinct patterns of protein distribution among the groups through comparative analysis of protein abundance. Comparing the common proteins among Exp *versus* Emb, 21 exhibited differential abundance, all of which were overabundant in Exp (Fig. 7*B*, Supplementary file 4*A*). Comparing the common proteins among Exp *versus* Exp + Emb, two showed differential abundance, all of which were overabundant in Exp (Fig. 7*C*, Supplementary file 4*B*). Comparing the common proteins among Emb *versus* Exp + Emb, seven exhibited differential abundance, all of which were overabundant in Exp + Emb (Fig. 7*D*, Supplementary file 4*C*).

Among the top 30 most abundant proteins in Exp (Table 4) and Exp + Emb (Table 4) were proteins such as zinc finger protein 638 (ZNF638), inositol polyphosphate-5-phosphatase E (INPP5E), actins (ACTB, and ACTC1), histones (H4 and H2B), annexins 2 and tubulins (TUBB). In Exp + Emb are also present others like tubulins (TUBB4B and TUBA1D), annexins (A0A4W2CRW3, ANXA4, and ANXA5), and OVGP1. In Emb, the most abundant proteins also included INPP5E, ZNF638, albumin (ALB), and ACTB (Table 4).

**Table 4:** Top 30 most abundant proteins in conditioned media extracellular vesicles (CM-EVs) from (A) the culture of oviductal explants in the absence (Exp) or (B) presence (Exp + Emb) of 8- to 16-cell stage embryos, or (C) from 8- to 16-cell stage embryos cultured alone (Emb)

(A) Top 30 most abundant proteins in CM-EVs from Exp.				
Protein accession	Gene name	Peptides	Razor + unique peptides	Description
A0A4W2FD79	ZNF638	5	5	Zinc finger protein 638
E1BAU6	INPP5E	2	2	Inositol polyphosphate-5-phosphatase E
A0A4W2DYQ2	ACTB	35	35	Actin beta
Q3ZC07	ACTC1	34	13	Actin, alpha cardiac muscle 1
Q3SYR8	JCHAIN	3	3	Immunoglobulin J chain
P62803	P62803	17	17	Histone H4
A0A3Q1M4X6	SMUG1	2	2	Single-strand selective monofunctional uracil DNA glycosylase
A0A4W2HPP0	–	23	23	Uncharacterized protein
A0A4W2HHA6	–	15	15	Histone H2B
A0A4W2G1A4	KRT76	9	2	Keratin, type II cytoskeletal 2 oral-like
A0A3Q1MDT7	–	14	3	Histone H4
A0A4W2H221	FGA	56	56	Fibrinogen alpha chain
A0A4W2CGL9	SMC3	20	20	Chondroitin sulfate proteoglycan 6
F1MZ85	VCAN	25	25	Versican core protein
A0A4W2HR21	SPTAN1	228	228	Spectrin alpha, non-erythrocytic 1
A0A4W2DVZ1	TUBB	28	28	Tubulin beta chain
F1MD77	LAMC1	54	54	Laminin subunit gamma 1
F1MGU7	FGG	31	31	Fibrinogen gamma-B chain
A0A4W2CVQ1	ANXA2	43	43	Annexin
A0A4W2III8	COL15A1	21	21	Collagen type XV alpha 1 chain
F6S1Q0	KRT18	44	44	Keratin 18
A0A452DJ66	TUBA1D	33	33	Tubulin alpha chain
A0A4W2CN44	DES	54	48	Desmin
P23805	CGN1	13	13	Conglutinin
F1MYC9	SPTBN1	199	199	Spectrin beta chain
A0A3Q1MPS4	ACTN1	72	72	Alpha-actinin-1
F1MNT4	LAMB1	60	60	Laminin subunit beta 1
P10096	GAPDH	28	28	Glyceraldehyde-3-phosphate dehydrogenase
A0A4W2BY01	VCL	65	65	Metavinculin
Q3MHM5	TUBB4B	27	7	Tubulin beta-4B chain

(B) Top 30 most abundant proteins in CM-EVs from Exp + Emb.				
Protein accession	Gene name	Peptides	Razor + unique peptides	Description
A0A4W2FD79	ZNF638	5	5	Zinc finger protein 638
E1BAU6	INPP5E	2	2	Inositol polyphosphate-5-phosphatase E
A0A4W2DYQ2	ACTB	35	35	Actin beta
A0A4W2G1A4	KRT76	9	2	Keratin, type II cytoskeletal 2 oral-like
A0A4W2HPP0	–	23	23	Uncharacterized protein
Q28042	OVGP1	44	44	Oviduct-specific glycoprotein (Fragment)
P62803	P62803	17	17	Histone H4
Q3SYR8	JCHAIN	3	3	Immunoglobulin J chain
A0A4W2HHA6	–	15	15	Histone H2B
Q3MHM5	TUBB4B	27	7	Tubulin beta-4B chain
A0A4W2DVZ1	TUBB	28	28	Tubulin beta chain
A0A4W2CRW3	–	27	27	Annexin
A0A3Q1MDT7	–	17	17	Histone H4
Q3ZC07	ACTC1	34	13	Actin, alpha cardiac muscle 1
A0A4W2CVQ1	ANXA2	43	43	Annexin
A0A452DJ66	TUBA1D	33	33	Tubulin alpha chain
F1MZ85	VCAN	25	25	Versican core protein
A0A4W2H221	FGA	56	56	Fibrinogen alpha chain
A0A4W2CGL9	SMC3	20	20	Chondroitin sulfate proteoglycan 6
F6S1Q0	KRT18	44	44	Keratin 18
P10096	GAPDH	28	28	Glyceraldehyde-3-phosphate dehydrogenase
A0A4W2EXX8	RPS8	14	14	40S ribosomal protein S8
F1MGU7	FGG	31	31	Fibrinogen gamma-B chain
A0A4W2E8T3	CD109	58	58	CD109 molecule
A0A4W2III8	COL15A1	21	21	Collagen type XV alpha 1 chain
A0A4W2EMD8	RPS6	20	20	40S ribosomal protein S6
A0A3Q1MHG7	ANXA4	29	29	Annexin
A0A4W2H475	RPLP0	19	19	60S acidic ribosomal protein P0
A0A4W2IRI4	ANXA5	33	33	Annexin
F1MD77	LAMC1	54	54	Laminin subunit gamma 1

(C) Top 30 most abundant proteins in CM-EVs from Emb.				
Protein accession	Gene name	Peptides	Razor + unique peptides	Description
E1BAU6	INPP5E	2	2	Inositol polyphosphate-5-phosphatase E
A0A4W2FD79	ZNF638	5	5	Zinc finger protein 638
A0A4W2G1A4	KRT76	9	2	Keratin, type II cytoskeletal 2 oral-like
A0A4W2CGL9	SMC3	20	20	Chondroitin sulfate proteoglycan 6
A0A3Q1M4X6	SMUG1	2	2	Single-strand selective monofunctional uracil DNA glycosylase
F6S1Q0	KRT18	44	44	Keratin 18
A0A4W2D966	VPS13D	3	3	Vacuolar protein sorting 13 homolog D
A0A4W2GAK0	SYNE1	24	24	Spectrin repeat containing nuclear envelope protein 1
A0A4W2GW83	ALB	24	19	Albumin
A0A4W2ENX3	KRT75	19	5	Keratin 75
F1MU12	KRT8	56	48	Keratin, type II cytoskeletal 8
A0A4W2E085	SRSF3	10	10	Serine and arginine rich splicing factor 3
F1MF78	SYNE2	73	73	Spectrin repeat containing nuclear envelope protein 2
A0A4W2HEC9	RASA4B	28	28	RAS p21 protein activator 4B
A0A4W2HA98	CEP135	2	2	Centrosomal protein 135
A0A4W2DYQ2	ACTB	35	35	Actin beta
F1MIW8	DSG1	5	5	Desmoglein-1
A0A4W2H231	DSP	78	78	Desmoplakin
A0A3Q1M1M7	JUP	22	22	Junction plakoglobin
A0A4W2ECV0	AIDA	4	4	Axin interactor, dorsalization associated
Q0MRP5	LZ	2	2	Lysozyme
A0A3Q1MBQ7	DNAH5	50	2	Dynein axonemal heavy chain 5
F1MWJ0	CDSN	2	2	Corneodesmosin
V6F7W7	UBE4A	9	9	Ubiquitin conjugation factor E4 A
P08728	KRT19	42	32	Keratin, type I cytoskeletal 19
E1B8N6	LMNB1	29	28	Lamin B1
A0A4W2IP44	DNAI2	4	4	Dynein axonemal intermediate chain 2
A0A3Q1LVU1	FTH1	12	12	Ferritin
A0A4W2H672	MELTF	8	8	Melanotransferrin
A0A4W2BM61	PBRM1	2	2	Polybromo 1

Functional enrichment using the PANTHER database showed that the identified proteins are involved in different GO biological processes and pathways. The four proteins exclusive to Exp are mainly related to cellular structure (Supplemental Fig. S2*A*). The six proteins exclusive to Exp + Emb, i.e. only when there is an interaction between the embryo and the explant, are mainly related to cellular processes, including binding, molecular function regulator activity, and catalytic activity (Supplemental Fig. S2*B*).

The five proteins only present in Exp + Emb and Emb, but not in Exp, are primarily carrier proteins and proteins associated with cellular processes, and biological regulation and participate in pathways such as the FAS signaling (Supplemental Fig. S2*C*). Most of the 452 proteins only present in Exp and Exp + Emb, but not in Emb, participate in cellular processes, metabolic processes, and biological regulation (Supplemental Fig. S2*D*). They are involved in pathways such as the integrin signaling pathway, which includes extracellular matrix (ECM) components (COL14A1, COL1A1, COL1A2, COL4A1, LAMA2, and LAMC1), cellular adhesion molecules (ITGA2, ITGA3, and ITGB1), cytoskeleton (ΑSMA, ACTC1, RAC1, and RHOA), and integrins (ITGA3, ITGB1, and ITGA2). Most of the 17 proteins only present in Exp and Emb, but not in Exp + Emb, are related to metabolic and cellular processes, including cell communication, cell adhesion, and motility. They are also involved in pathways such as Integrin signaling (Supplemental Fig. S2*E*). Proteins common to all groups are mainly structure-related proteins, involved in pathways such as the integrin signaling pathway, inflammation, cytoskeletal regulation, Wnt signaling, and the PI3K pathway (Supplemental Fig. S2*F*).

Functional enrichment analyses using the PANTHER database were also conducted with DAP. When comparing CM-EVs from Exp *versus* Exp + Emb, we identified two DAPs, both more abundant in Exp + Emb. Only PSMB8 is classified by the PANTHER database. This protein is a modifying enzyme with catalytic activity that participates in metabolic processes. No pathway hits were identified (Supplemental Fig. S3*A*). When comparing CM-EVs from Exp *versus* Emb we identified 21 DAP, all more abundant in Exp. Most of these proteins are involved in cellular processes, biological regulation, and metabolic processes. Additionally, they are also components of pathways such as the Wnt signaling pathway (Supplemental Fig. S3*B*). When comparing CM-EVs from Emb *versus* Exp + Emb, we identified seven DAPs, all more abundant in Exp + Emb. Most of them are primarily structure-related proteins, exhibiting binding activity and participating in pathways such as cytoskeletal regulation (Supplemental Fig. S3*C*).

#### Comparison of *in vivo* Model and *in vitro* Model

**Fig. 8:**
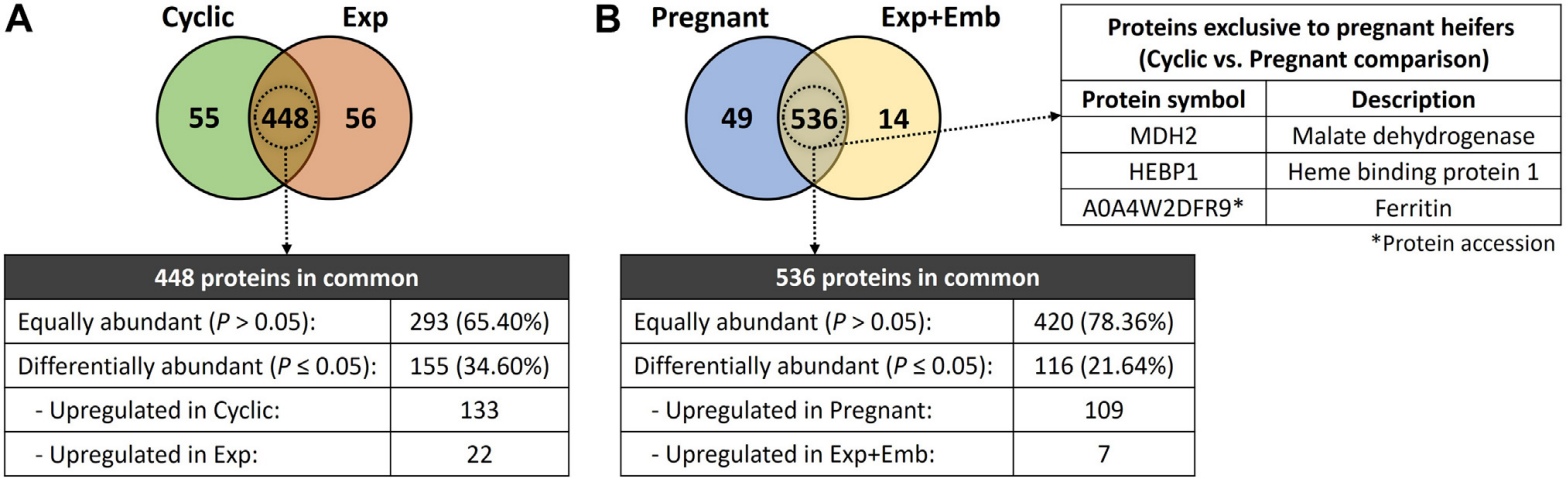
**Number of proteins identified between the *in vivo* and the *in vitro* model.***A*, Venn diagram representing the number of proteins associated with oviductal fluid extracellular vesicles (OF-EVs) from Cyclic heifers *versus* conditioned media extracellular vesicles (CM-EVs) from oviductal explants cultured alone *in vitro* (Exp). *B*, Venn diagram representing the number of proteins associated with OF-EVs from Pregnant heifers *versus* CM-EVs from oviductal explants cultured with 8- to 16-cell embryos (Exp + Emb) *in vitro*, and three proteins (MDH2, HEBP1, and A0A4W2DFR9) unique to the OF-EVs of Pregnant heifers also identified in CM-EVs from Exp + Emb.

To identify the differences and similarities among the *in vivo* and *in vitro* models, we compared the OF-EVs from Cyclic heifers with CM-EVs from Exp and OF-EVs from Pregnant heifers with CM-EVs from Exp + Emb. When comparing OF-EVs from Cyclic heifers and CM-EVs from Exp (Fig. 8*A*), we identified 55 proteins unique to Cyclic, 56 unique to Exp, and 448 common proteins. Of these, 155 (34.60%) were DAPs. Comparing OF-EVs from Pregnant heifers and CM-EVs from Exp + Emb (Fig. 8*B*), we identified 49 proteins unique to Pregnant, 14 unique to Exp + Emb, and 536 common. Of these, 116 (21.64%) were DAPs.

Functional enrichment using PANTHER highlighted different GO biological processes when comparing proteins identified in CM-EVs from Exp and proteins identified in OF-EVs from Cyclic heifers. Proteins unique to OF-EVs from Cyclic heifers are mainly protein-modifying enzymes and transporters related to cellular and metabolic processes and with binding and catalytic activity (Supplemental Fig. S4*A*). Proteins unique to CM-EVs from Exp are mainly metabolite interconversion enzymes and protein-modifying enzymes, related to cellular processes, biological regulation, and with binding and catalytic activity (Supplemental Fig. S4*B*). Equally abundant proteins (EAPs) are mainly metabolite interconversion enzyme and cytoskeletal protein related to cellular, metabolic, and biological processes, also related with binding, catalytic and ATP-dependent activities (Supplemental Fig. S4*C*). DAPs are mainly translational protein and protein-binding activity modulators related to cellular and metabolic processes with binding, structural, and catalytic activities (Supplemental Fig. S4*D*).

Additionally, we performed functional enrichment using PANTHER with proteins identified in CM-EVs from Exp + Emb and proteins identified in OF-EVs from Pregnant heifers. Proteins unique to OF-EVs from Pregnant heifers are mainly scaffold/adaptor proteins, membrane traffic proteins, and protein modifying enzymes. Moreover, they are related to cellular process and biological regulation (Supplemental Fig. S5*A*). Proteins unique to CM-EVs from Exp + Emb are mainly metabolite interconversion enzymes with cellular and metabolic processes, and catalytic activity (Supplemental Fig. S5*B*). EAPs are mainly cytoskeletal proteins, translational proteins, and metabolite interconversion enzymes. Besides, they are related to cellular, metabolic, and biological processes, with binding, catalytic, and structural activities (Supplemental Fig. S5*C*). DAPs are mainly protein-binding activity modulators and metabolite interconversion enzymes. Additionally, they are related to cellular and biological regulation and response to stimulus (Supplemental Fig. S5*D*).

**Fig. 9:**
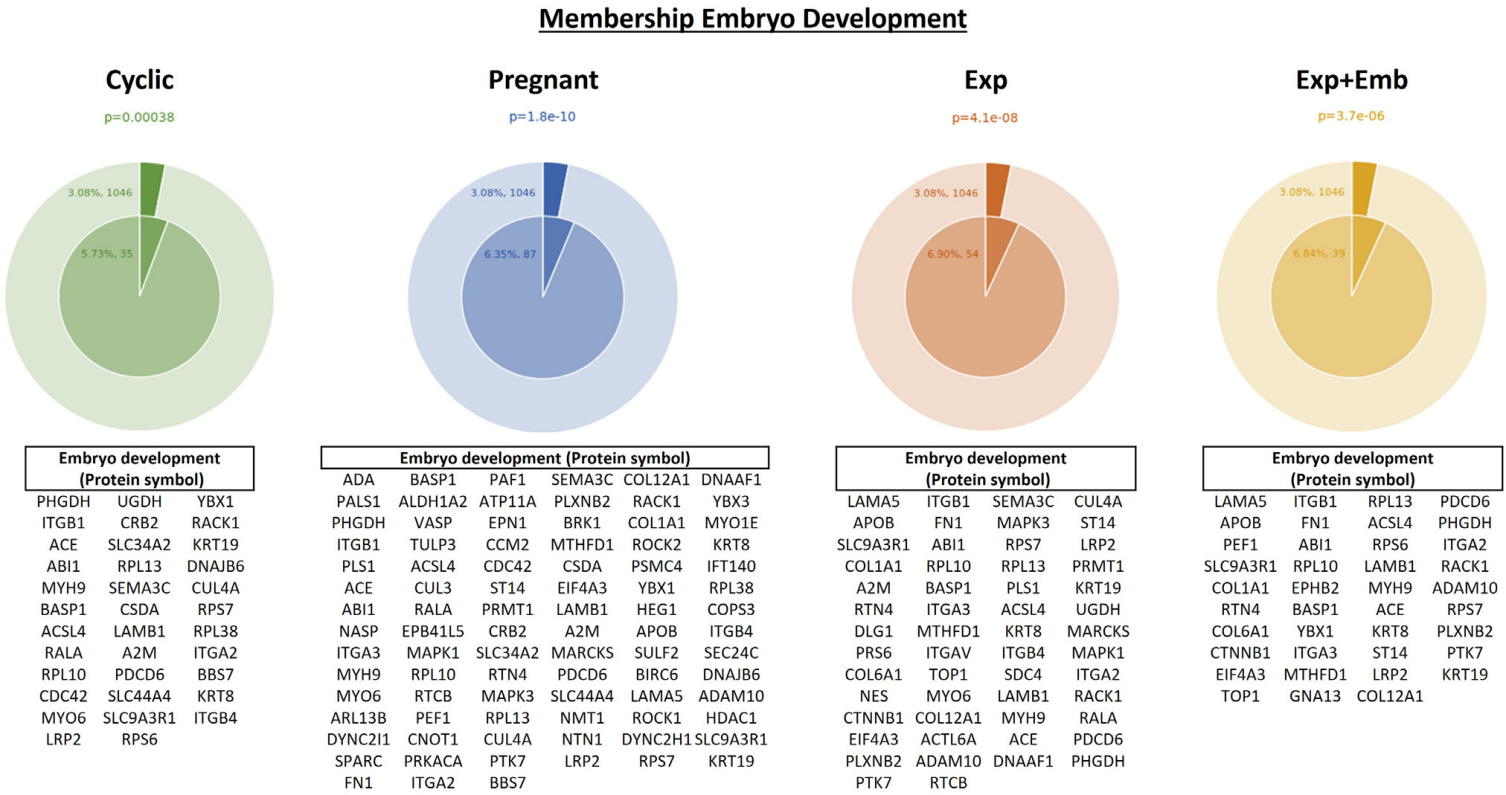
**Functional membership analysis for proteins matching with “embryo development” term.** Proteins identified in the oviductal fluid extracellular vesicles from Cyclic (*green* pie chart) and Pregnant Heifers (*blue* pie chart) and in the conditioned media extracellular vesicles from explants cultured alone (Exp; *orange* pie chart) and explants cocultured with embryos (Exp + Emb; *yellow* pie chart). The *outer* pie chart illustrates the count and proportion of proteins within the background dataset that are affiliated with the “embryo development” membership, whereas the inner pie chart presents the count and proportion of proteins in the specific input gene list associated with this membership. The *p*-value positioned above the pie charts indicates a statistically significant enrichment of the membership across all groups.

Furthermore, functional membership analysis for proteins matching the “embryo development” term was conducted with the proteins identified *in vivo* and *in vitro* models (Fig. 9). OF-EVs from Pregnant heifers were the ones with a higher number of proteins associated with embryo development (36), while in OF-EVs from Cyclic heifers 35 proteins related to “embryo development” were identified. While *in vitro*, 54 proteins related to embryo development were identified in the CM-EVs from Exp and 39 from Exp + Emb.

## Discussion

To our knowledge, this is the first study reporting proteomic characterization of embryo-induced alterations in bovine oviductal EVs around day 3.5 of pregnancy, both *in vivo* and *in vitro*. Results indicate that embryo-maternal communication, mediated by EVs, is initiated in the oviduct. OF-EVs from cyclic and pregnant heifers exhibit different protein profiles, as do the CM-EVs from oviductal explants cultured in the absence or presence of 8- to 16-cell stage embryos. Although the effect of sperm cannot be entirely ruled out in the Pregnant group, results indicate that the embryo can induce changes in the protein profile of OF-EVs, a capacity further supported by the results using the *in vitro* model, from which sperm were absent. Differentially abundant proteins in pregnant heifers were mainly related to genome activation, DNA repair, embryonic cell differentiation, migration, and immune tolerance. On the other hand, proteins identified during the interaction between the oviduct and the embryo *in vitro* were linked to several functions, including immune tolerance, structural activity, binding, and cytoskeleton regulation. Moreover, *in vivo* and *in vitro* EVs demonstrate marked differences in their qualitative and quantitative protein composition.

### Oviductal and Embryonic EVs

*In vivo*, changes in OF-EV content, including proteins, mRNAs, small ncRNAs (19), and miRNAs (20), have been explored across the bovine estrous cycle. Unlike these previous investigations, this study characterized the EV protein content at an exact time using synchronized heifers instead of tissues sourced randomly from abattoirs. Recently, changes in the OF-EV miRNA content have also been described in pregnant cows (8). Building on this, we characterized the protein content of OF-EVs from cyclic and pregnant heifers, focusing on embryo-maternal communication and further comparing it with our *in vitro* model through explants from the same cyclic heifers.

*O*viductal EVs from BOEC were previously detected by Almiñana *et al*. (17) and Lopera-Vásquez *et al*. (18). In contrast to these studies, which used 2D monolayer cultures, we have used oviductal explants to investigate embryo-maternal communication *in vitro* as explants preserve both cellular and extracellular architecture, facilitating communication between different populations of oviductal epithelial cells. Moreover, embryonic EVs have mainly been identified in media conditioned by bovine blastocysts *in vitro* (37, 38), with most studies focusing on the EV miRNA profile under different oxygen tensions (37) or related to embryo viability (38). Recently, the miRNA content of EVs originating from bovine 8-cell embryos has also been studied (39). In contrast, we have focused on the role of embryonic EVs in embryo-maternal communication, and for the first time to our knowledge, we describe the protein profile of EVs in the bovine oviduct in response to the presence of an 8 to 16 cell embryo.

### *In vivo*: Identified Proteins

Among the major abundant proteins in OF-EVs from cyclic and pregnant heifers are proteins such as the OVGP1, annexins (ANXA2, ANXA4, ANXA5, and ANXA11), and ribosomal proteins. OVGP1 is an oviduct-specific glycoprotein, known for its role in modulating sperm-zona pellucida interaction during fertilization (40) and supporting embryonic development (41). Our findings are consistent with previous reports, as OVGP1 was also one of the most abundant proteins in the OF of sheep (42), pig (36), and bovine OF-EVs (17). Importantly, OVGP1 is an embryo-interacting protein that can pass through the zona pellucida and be taken up by the embryo (43), with its enriched presence in EVs indicating a potential mechanism for its delivery via these vesicles. Reinforcing its function in supporting embryo development, oviductin is among the most overabundant proteins in bovine embryos following *in vivo* development, which are known for their higher quality than those *in vitro* derived (44).

Consistent with previous reports, annexins 2, 4, and 5 have been detected among the most abundant proteins in the OF (43) and ANXA2 in OF-EVs (17). Annexins are involved in several processes, including exosome trafficking, membrane repair, cellular proliferation, apoptosis and migration, and inflammatory responses at the maternal-fetal interface in several species (45). In mares, ANXA2 increased in the UF during pregnancy recognition (46), and ANXA4 is overabundant in the OF of pregnant mares (47). ANXA2 has been implicated in embryo attachment in mice (48) and humans (49), while ANXA4 may promote trophoblast invasion via the PI3K/Akt/eNOS pathway in humans (50). Recently, annexins 2, 4, and 5 have been identified among the most abundant embryo-interacting proteins in OF-treated bovine embryos (43), highlighting them as key players not only in embryo implantation and pregnancy but also in early bovine embryo-maternal interaction in the oviduct.

Ribosomal subunits in OF-EVs are directly linked to protein translation, impacting various biological processes, including embryo development (51). Notably, RPS16 and RPL6 have also been identified in bovine OF and shown to interact with embryos during the 4- to 6-cell stage (43). Also, mRNAs encoding ribosomal proteins are the most abundant protein-coding RNAs in bovine OF-EVs (19). Interestingly, studies in cancer suggest that EVs carry specific mRNAs, which are translated into proteins that support ribosomal functions, facilitating the translation of other EV mRNAs in the recipient cell (52). Therefore, we speculate that the maternal delivery of ribosomal components through OF-EVs may be particularly important to support the initial embryonic cell cycles, as, during this stage, the embryo relies on the oocyte pool of ribosomes for protein synthesis until the activation of embryonic ribosomal RNA (rRNA) genes during major EGA (53).

### *In vivo*: Proteins Exclusive to OF-EVs From Pregnant Heifers

EVs are known to be present in bovine OF and have functional impacts on embryo development (8, 16, 17, 18). Our findings indicate that specific proteins exclusive to EVs from pregnant heifers are potentially mediating critical processes for early embryo development, including EGA transition (CUL1, DHX15, and PRMT1), DNA repair process (UBA2 and RAD50) and cell differentiation (HEBP1 and RPS21). The absence of these proteins in OF-EVs from cyclic heifers is consistent with previous findings (17, 19), which did not detect these proteins in OF-EVs from cyclic animals, regardless of the estrous cycle stage. The consistent absence of these proteins in cyclic OF-EVs, along with their exclusivity in OF-EVs from pregnant animals in our study, reinforces their potential involvement in early embryo-maternal interaction through EVs within the oviduct.

In cattle, EGA occurs within the oviduct at the 8- to 16-cell stage (10), which corresponds to the period during which this study was conducted. CUL1 is synthesized during early bovine embryonic development, with increased abundance at 4-cell and 8-cell stages, and is potentially involved with the degradation of maternal proteins during EGA (54). Supporting its importance, null mouse embryos show early embryonic lethality and disrupted regulation of the cell cycle regulatory protein cyclin E (55). Additionally, in equine embryos, DHX15 gene expression increases from the 8-cell stage, peaks at the 16-cell stage, and decreases continuously after EGA (56). DHX15 is also expressed at the 8-cell stage of bovine embryos (10) and is involved in RNA metabolism, ribosome biogenesis, transcription initiation, and differentiation processes (57). Similarly, PRMT1, a histone modifier protein that leads to transcription activation, peaks at the 4-cell stage and undergoes down-regulation after EGA in bovine embryos (58). PRMT1 is crucial for mouse embryogenesis, as PRMT1−/− mice fail to develop beyond day 6.5, likely due to its role in regulating DNA damage checkpoint control, genome integrity, and cell proliferation (59). Therefore, the exclusive presence of CUL1, DHX15, and PRMT1 in OF-EVs from pregnant animals, along with their embryonic expression patterns, highlights their involvement in EGA and suggests that maternal EVs may provide molecular support for early embryonic development.

The proteins exclusive to OF-EVs from pregnant heifers are also related to DNA damage detection and repair. One such critical protein, RAD50, exhibited high activation during EGA in mouse and pig embryos (60). It has also been proposed as a biomarker of developmental competence in early bovine and mouse embryos (61, 62), and its disruption leads to embryonic stem cell lethality and abnormal embryonic development in mice (63). UBA2 regulates protein structure, stability, function, and localization, impacting numerous physiological functions, including DNA repair (64). UBA2 mRNA levels are elevated in the early-stage porcine embryo, aligning with the EGA period (65). The same study observed that induced DNA damage decreased UBA2 expression in day 4 embryos, indicating that the regulation of UBA2 may have relevant roles in the repair of damaged DNA (65). Moreover, supplementing UBA2 in the IVC medium increased embryo development in pigs, suggesting its regulation of normal embryo development through preserving genome stability (66). Therefore, as embryos deficient in DNA repair enzymes are typically nonviable (28), we hypothesize that EVs act as a delivery system for essential DNA repair components, such as UBA2 and RAD50, especially during the early stages when the embryo's genome is still activating.

### *In vivo*: Proteins More Abundant in OF-EVs From Pregnant Than Cyclic Heifers

The overexpression of MDH1, IDH1, and TPI1 potentially mediate energy metabolism, consistent with the embryonic metabolic changes within the oviduct, shifting from pyruvate utilization to increased glucose oxidation (3). Similarly, TPI1 has been detected in higher concentrations in the UF of pregnant cattle (67) and IDH1 exhibits elevated expression in the bovine endometrium (68) and in the UF during early pregnancy (67). MDH1 and IDH1 are involved in the Krebs cycle and NADH metabolic pathways (69). MDH1 serves as a key regulator of carbohydrate metabolism, determining developmental progression in the pre-implantation embryo, with its ablation leading to decreased embryo development in mice (70). Furthermore, TPI1 transcript, critical for glycolysis and efficient ATP production (71), has been identified as a biomarker for blastocyst competence (72), and it was found to be downregulated under oxidative stress in bovine embryos (73). Based on that, we propose that the overabundance of these proteins in OF-EVs from pregnant heifers may support the changes in embryonic metabolism in the oviduct. Additionally, we hypothesized that OF-EVs from pregnant animals would aid in vitro-produced embryos in managing the metabolic stress associated with the *in vitro* environment.

Additionally, overabundant proteins in OF-EVs from pregnant heifers, such as TAGLN2 and ITGB1, may play a critical role in embryogenesis and implantation. Although TAGLN2 and ITGB1 have been previously identified in the OF (74) and in OF-EV content from both *in vitro* and *in vivo* origins (17) in cyclic cows, our results indicate that their abundance may reflect the presence of the embryo. TAGLN2 is highly expressed in murine trophoblasts, where it regulates trophoblast invasion and adhesion during implantation by promoting actin polymerization (47). Similarly, ITGB1 exhibits continuous expression throughout pre-implantation bovine embryo development (75) and is essential for cell-cell adhesion, cell-extracellular matrix adhesion, and signal transduction, contributing to embryogenesis, and implantation processes (76). Notably, knockout studies in mice demonstrate that embryos lacking ITGB1 fail to implant due to impaired trophoblast migration and proliferation (77). Therefore, the overabundance of these proteins in OF-EVs from pregnant heifers suggests their involvement not only in embryogenic processes but also in paracrine communication between the oviduct and uterus, potentially contributing to the preparation of the endometrium for receiving the embryo.

### *In Vitro* Model

Oviduct explant models have been primarily employed to study equine and boar sperm attachment (78). Another study demonstrated that explants obtained by scraping the ampullary-isthmic region of the equine oviduct remain morphologically and functionally intact after 6 days of culture, indicating that the explants are a reliable model to investigate the interactions between the embryo and oviduct in horses (79). On the other hand, in cattle, explants have been predominantly utilized for studying the uterine environment. Specifically, endometrial explants have been employed as an *ex vivo* model to study immunity and inflammation (28), the effects of interferon tau (IFNT), conceptus origin (*in vivo versus in vitro*), conceptus sex (28), conceptus size (80) and embryo maternal interaction (81). These studies also demonstrated that a 6-h culture period is sufficient to induce alterations in response to the presence of the embryo while preserving the structural and functional integrity of the explants (82). The current study demonstrated that CM-EVs from oviductal Exp and Exp + Emb have a distinct protein profile, reflecting the embryo-maternal interactions between the 8- to 16-cell stage embryo and the oviductal explant *in vitro*.

### *In Vitro*: Exclusive in CM-EVs From Exp + Emb

Similar to the *in vivo* model, several proteins were found exclusively in the interaction between the embryo and maternal tissue *in vitro*. Reinforcing the potential impact of the embryo on the presence of these proteins in the bovine oviductal environment, PRKAB1, UPK3BL2, PTPA, PTPRD, and TOM1L1 have not been previously identified in OF-EVs across the estrous cycle (19), nor in the content of OF-EVs from both *in vitro* and *in vivo* origins (17) in cyclic cows. Interestingly, compared to our *in vivo* model, four of these proteins (PRKAB1, PTPA, PTPRD, and TOM1L1) were considered present only in OF-EVs from pregnant animals. Although these proteins could potentially participate in metabolic energy processes, cell growth, differentiation, and the mitotic cycle of the embryo and oviductal epithelial cells of pregnant animals, further studies are needed to investigate their functions in these contexts. On the other hand, S100A11 has been previously detected in oviductal EVs from both *in vivo* and *in vitro* origins (17) as well as in the OF around the time of ovulation (74). Additionally, S100A11 transcript is also present in porcine OF-EVs (83), and when these EVs are introduced into the *in vitro* embryo culture, an elevated expression of S100A11 was detected in the produced embryos, indicating that OF-EVs are modulating the embryonic transcriptome (84).

S100A11, a member of the S100 protein family, is reported to mediate immunotolerance during pregnancy, enhance endometrial receptivity, and facilitate embryo adhesion by activating calcium-triggered signaling pathways and promoting the expression of genes such as EGFR, IL-15, and LIF (85). S100A11 could also be critical in maintaining the equilibrium of Th1 (pro-inflammatory) and Th2 (anti-inflammatory) cytokines, which is crucial for immune tolerance to the semi-allogeneic embryo *in vivo* and *in vitro*. Downregulation of endometrial S100A11 may contribute to reproductive failure in mice by reducing embryo implantation rates and adversely affecting the expression of factors associated with endometrial receptivity (86). In humans, S100A11 is also detected in the endometrium and is associated with adverse immune as protein abundance of S100A11 was notably reduced in the endometrium of women who experienced pregnancy failure compared to those with favorable pregnancy outcomes (86). Additionally, it has been shown that the downregulation of S100A11 from endometrial cells leads to a significant reduction in Th2 cytokines and a substantial increase in Th1 cytokines (87). Therefore, these findings suggest that S100A11 may play a role in immunomodulation within the oviduct by affecting the balance between Th1 and Th2 cytokines.

### *In Vitro*: CM-EVS From Exp *versus* Exp + Emb

Several proteins were identified in Exp and Exp + Emb as related to early embryo development (ADAM10, ANPEP, ATP1A1, ATP1B1, CLTC, MACROH2A1, RACK1, and RPSA). However, only two (A0A4W2DFR9 and PSMB8) were differentially abundant, with PSMB8 noteworthy for its potential role in immunomodulation and regulation of endometrial cell activity during elongation. PSMB8, a protein-modifying enzyme, was previously identified in the OF of cyclic cows around ovulation (74). Additionally, its transcripts were identified in the uterine endometrium on day 16 of bovine pregnancy (88) and in the pig myometrium on day 15 of pregnancy (89). In goats, PSMB8 within UF-EVs may contribute to establishing a receptive endometrium and promoting embryo implantation by regulating cell migration, proliferation, and apoptosis through modulation of the ERK1/2 and PI3K/AKT signaling pathways (90, 91). Moreover, PSMB8's function extends to the immune system as the down-regulation of antigen processing machinery, which includes PSMB8, may impact the MHC-I pathway and the generation of antigenic peptides (92), thereby influencing immune tolerance to the embryo. This highlights that PSMB8 within EVs might influence both cellular regulation and antigen presentation, processes essential for pregnancy success (93). Nonetheless, further research is needed to investigate the PSMB8 protein profile in oviductal cells and its role in immunomodulation during pre-implantation embryo development in the oviduct.

### *In Vitro*: CM-EVs From Exp + Emb *versus* Emb

Five proteins were only present in CM-EVs from Exp + Emb and Emb (TSN, TTC26, MELTF, LMNB1, and MUC16), and seven (TPM1, ACTB, ANXA6, PSMA6, SPTBN1, SPTAN1, and ACTC1) overabundant in Exp + Emb. Although identified in embryos and in the maternal environment, most of these proteins lack clearly defined reproductive functions but are associated with structural activity, binding, and cytoskeletal regulation, as indicated by PANTHER analysis. For example, ANXA6 and SPTBN1 are cytoskeletal protein binding factors likely involved in early bovine embryo differentiation processes (94, 95). While MUC16, a membrane-associated mucin, helps form a non-adhesive barrier, the reduction of which in the endometrial luminal epithelium during the receptive phase enhances embryo adhesion and facilitates conceptus attachment (96), and contributes to endometrial remodeling in the bovine endometrium (97). Hence, within the oviductal environment, the presence of MUC16 may be linked to preventing embryo adhesion to the oviductal epithelium, facilitating their transit.

### *In Vitro*: CM-EVs From Exp *versus* Emb

All DAPs in CM-EVs identified between Exp and Emb were overabundant in the Exp group, including proteins such as GAPDH, ANXA2, and ANXA4. GAPDH is present in OF-EVs from both pregnant and cyclic animals, exhibiting no significant difference between them. Moreover, ANXA2 and ANXA4, among the most abundant proteins in OF-EVs in both cyclic and pregnant heifers, are implicated in membrane trafficking and fusion processes that are frequently seen in exosomes (98). The overabundance of these proteins in the CM-EVs from Exp suggests that they could be more relevant for intercellular communication, physiological functions, or specific signaling pathways of oviductal cells compared to those of the embryo, as they are present in both, but in higher quantities in the EVs from oviductal explants.

Moreover, 17 proteins were only present in Exp and Emb, but not in Exp + Emb. We hypothesize that these proteins undergo modifications during *in vitro* embryo-oviduct interaction, as they are present in separate samples but absent in co-culture. However, compared to the *in vivo* model, 10 proteins (AP3D1, PBRM1, RPL36, PMM2, DDX31, PARP1, DNAI2, ITGB4, SYNE2, and USP9X) are similarly abundant in EVs from the pregnant group and cyclic group, while seven proteins (PLOD1, SYNE1, COG5, TPD52, SERPINB13, SYNM, and CCT5) appear exclusively in EVs from Emb and Exp *in vitro*. The results suggest that oviduct-embryo interactions may unfold distinctly between *in vitro* and *in vivo* models, as evidenced by distinct EV protein patterns, with differences and similarities to be discussed further.

### In Vivo versus In Vitro

*In vivo*- and *in vitro*-derived EVs exhibit distinct qualitative and quantitative protein contents, both when comparing EVs that have not had contact with the embryo (Cyclic and Exp) and those that have undergone embryo-oviduct interaction (Pregnant and Exp + Emb). Between pregnant and Exp + Emb groups, involving maternal-embryonic communication, 420 EAPs were shared, indicating that most of the proteins (78.36%) were common to both *in vivo* and *in vitro* models and highlighting their importance in cell function independently of the model. Indeed, as indicated by the PANTHER database, these proteins relate to cellular processes and include annexins (ANXA2, ANXA4, ANXA5), mucins (MUC4), adhesion proteins, motility regulators (ACTB, ACTR3), cell adhesion (AGRN, CD47, TLN1, ITGA3), communication (ADAM10, ATP1A1, ATP1B1, RACK1, RPSA), and differentiation (LAMC1, ANPEP, CLTC, MACROH2A1, HSP90AA1). Furthermore, YBX1, a transcription factor that mediates maternal mRNA decay during EGA, was also common in both models (99). Additionally, three proteins related to cell metabolism (MDH2, HEBP1) exclusively identified in the OF-EVs of pregnant heifers were also detected in CM-EVs from Exp + Emb, indicating that some proteins exclusive to pregnancy remain present even when embryos are co-cultured *in vitro* with explant. These findings indicate that most of the identified proteins do not vary across systems, suggesting their importance in biological processes both *in vivo* and *in vitro*.

On the other hand, 49 proteins were present exclusively in OF-EVs from pregnant heifers, but not in CM-EVs from Exp + Emb, and may influence embryo development and impact on the maternal environment. For instance, SPARC, LTBP1, and ITGB1BP1 could be associated with the stimulation of endometrial receptivity by regulating non-invasive implantation in cattle (97, 100, 101). CENPE, a marker of developmental competence of rapidly cleaving bovine two-cell embryos (102), is essential to maintain chromosomal stability, with its knockout resulting in early developmental arrest in mice (103). Additionally, EPN1 and TULP3 regulate the pluripotency of mouse stem cells, and ablation of EPN1 impairs embryo development (104, 105). Besides, GAS1 plays a significant role in human embryonic development, orchestrating cell cycle, specification, and stem cell biology, all essential for successful embryo development (106). Furthermore, JAK3, a JAK/STAT signaling pathway component, participates in the signaling activation of many cytokine receptors, potentially regulating embryo cell survival and proliferation (107). Therefore, although many proteins are common between pregnant heifers and Exp + Emb, our results suggest that EVs from the *in vivo* environment may provide better support for embryo development.

Importantly, different studies have shown that EVs isolated from BOECs conditioned medium (18) or OF (16, 17) were internalized by the embryo and favored the development and quality of bovine *in vitro*-produced embryos. Recently, sequential use of EVs from OF and UF during bovine embryo IVC also improved embryo quality by lowering lipid contents (24). Moreover, EV effects on cell proliferation, as observed by increased cell number in blastocysts cultured with EVs, were also detected, suggesting that EVs may modulate embryo development through their content, including their protein cargo (24). Considering that EVs from the *in vivo* environment may provide better support for embryo development, we hypothesize that supplementation with OF-EVs from pregnant animals would induce a better effect during *in vitro* embryo development.

Indeed, OF-EVs from pregnant animals also exhibit a higher number of proteins associated with embryo development. Notably, out of the 87 proteins related to “embryo development” in the OF-EVs from pregnant heifers, 32 proteins were considered present only in this group. These include PALS1, PAF1, ROCK1, ROCK2, HDAC1, BIRC6, NASP, CNOT1, and PSMC4, which may play critical roles in pre-implantation embryo development. For instance, NASP is implicated in cell division regulation and transcriptional control, particularly in fast-developing zygotes associated with higher blastocyst rates (108). Additionally, PAF1 is essential for regulating stem cell pluripotency by positively influencing the expression of key pluripotency genes like OCT4 and NANOG (109). Complementing this, HDAC1 regulates histone deacetylation, development, and gene expression, especially around EGA at the eight-cell stage in pre-implantation mouse embryos (110). PALS1, a central component of the cell polarity network and an upstream modulator of the Hippo pathway may regulate blastomeres' polarity during mouse embryonic development (111). Similarly, ROCK2 may also promote blastocyst formation, while ROCK1 primarily regulates morula compaction (112), with their mRNA abundance in BOECs influenced by the presence of the bovine early embryo (113), highlighting their potential role in early pregnancy. Further, BIRC6 is involved in apoptosis regulation, and its depletion leads to a lower proportion of 8-cell bovine embryos, followed by embryonic arrest during early pre-implantation embryo development (114). Finally, the PSMC4, a component of the ATPases proteasome complex, is crucial for normal mouse embryonic development, as its knockout leads to embryo arrest after day 3.5 of pregnancy (114). Therefore, although maternal-embryonic communication can be replicated *in vitro*, CM-EVs from Exp + Emb lack critical proteins present in OF-EVs from pregnant heifers, which can be delivered to the embryo and influence essential processes for proper early development.

## Conclusion

In conclusion, the study characterized for the first time a specific protein signature in OF-EVs from Pregnant animals, which is likely due to the interactions established between the maternal oviduct and the embryo. Differentially abundant proteins in Pregnant heifers are mainly related to genome activation, DNA repair, embryonic cell differentiation, migration, and immune tolerance. Additionally, the proteins identified when there is an interaction between the oviduct and the embryo *in vitro* are associated with immune tolerance, structural activity, binding, and cytoskeletal regulation. As evidenced by our results, there are notable qualitative and quantitative differences in the protein profile of EVs derived from *in vivo* and *in vitro* origins. While *in vitro* models provide a valuable starting point for investigating maternal-embryonic communication, it is crucial to acknowledge that oviduct explants and embryos encounter unique challenges and conditions *in vitro*, leading to distinct stimuli and signaling compared to the reproductive tract *in vivo*. In summary, alterations in protein content within EVs resulting from maternal-embryonic communication are observed *in vivo* and *in vitro*, highlighting the initiation of embryo-maternal dialogue within the oviduct, potentially facilitated via EVs and their protein cargo.

## Ethics Approval

All experimental procedures involving animals were approved by the Animal Research Ethics Committee of University College Dublin and licensed by the Health Products Regulatory Authority, Ireland, in accordance with Statutory Instrument No. 543 of 2012 under Directive 2010/63/EU on the Protection of Animals used for Scientific Purposes.

## Data Availability

The mass spectrometry proteomics data have been deposited to the ProteomeXchange Consortium (https://proteomecentral.proteomexchange.org) via the iProX partner repository (115, 116) with the dataset identifier PXD053670.

## Supplemental data

This article contains supplemental data.

## Conflicts of interest

The authors declare that they have no conflicts of interest with the contents of this article.
