# Supplementary material for: Embryo-Induced Changes in the Protein Profile of Bovine Oviductal Extracellular Vesicles
Source: Mol Cell Proteomics. 2025 Feb 28;24(4):100935. doi: 10.1016/j.mcpro.2025.100935 (PMC11994978; doi:10.1016/j.mcpro.2025.100935)

Figure S1. Functional Enrichment of the Proteins Less Abundant in Oviductal Fluid Extracellular Vesicles of Pregnant Heifers. (A) protein class, (B) gene ontology, and (C) pathways identified using the PANTHER 18.0 Classification System (https://pantherdb.org/). Darker bars indicate the number of genes associated with each category name, while lighter bars represent the percentage of these genes relative to the total number of genes in that category.

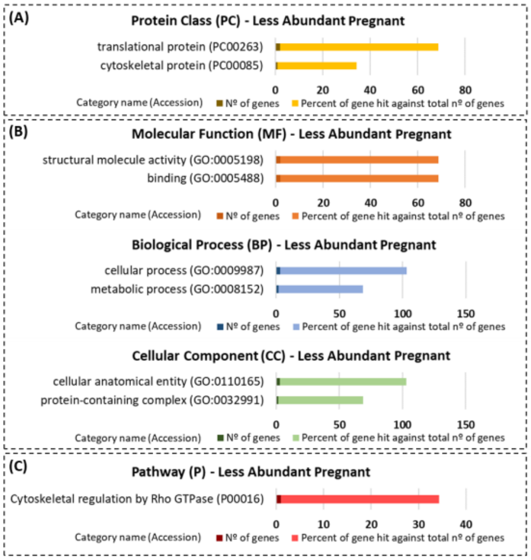

Figure S2. Functional Enrichment of the Proteins Identified in Conditioned Media Extracellular Vesicles (CM-EVs) *in Vitro*. (A) Four proteins were exclusive to CM-EVs from explants cultured alone (Exp). (B) Six proteins were exclusive to CM-EVs from explants co-cultured with embryos (Exp+Emb). (C) Five proteins were only present in CM-EVs from Exp+Emb and embryos cultured alone (Emb). (D) Four hundred fifty-two proteins were only present in CM-EVs from Exp and Exp+Emb. (E) Seventeen proteins were only present in CM-EVs from Exp and Emb. (F) Eighty-one proteins were common in Exp, Exp+Emb, and Emb. Protein class, gene ontology, and pathways were identified using the PANTHER 18.0 Classification System (https://pantherdb.org/). Darker bars indicate the number of genes associated with each category name, while lighter bars represent the percentage of these genes relative to the total number of genes in that category.

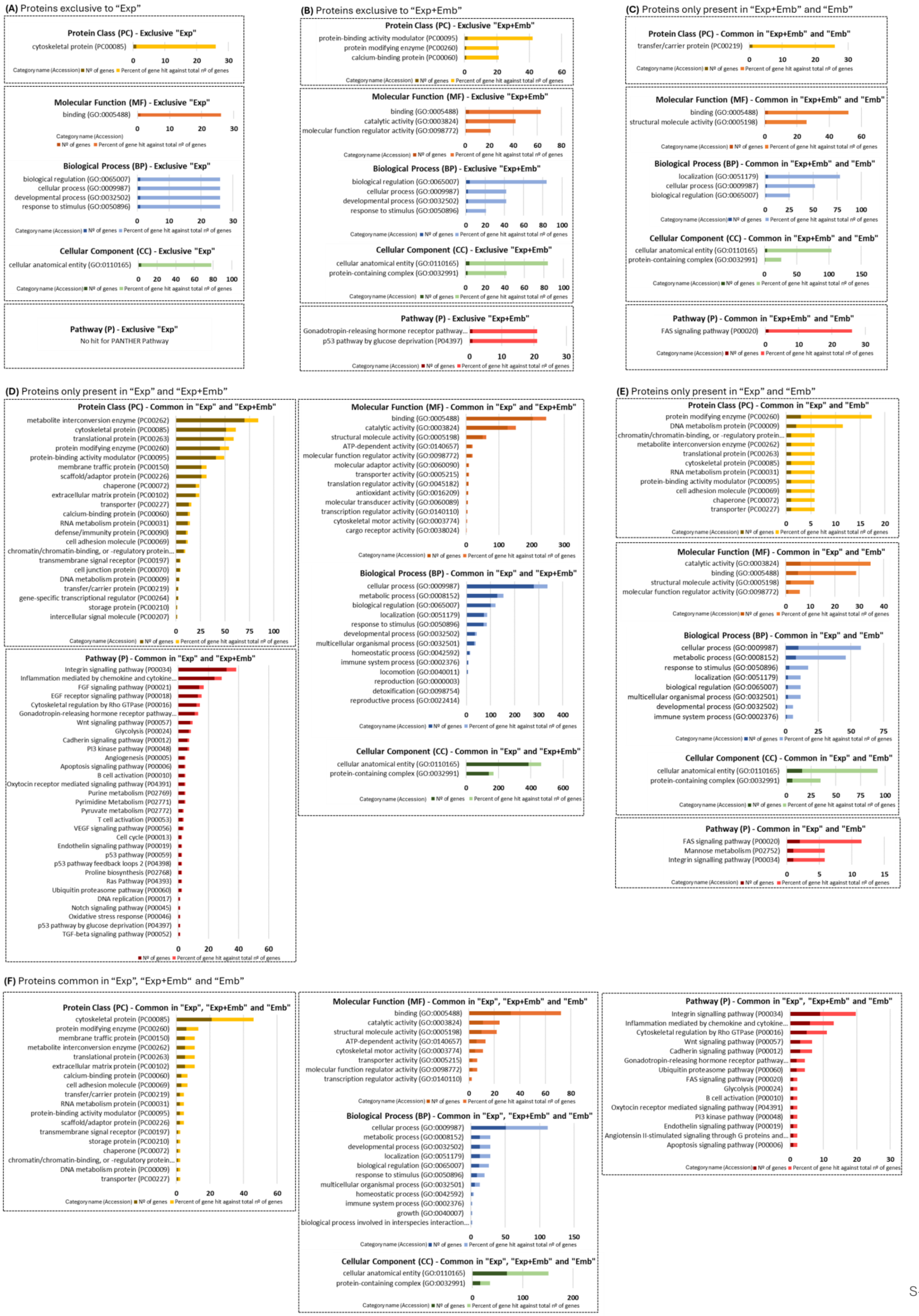

Figure S3. Functional Enrichment of the Differentially Abundant Proteins Identified in Conditioned Media Extracellular Vesicles (CM-EVs) *in Vitro*. (A) Two proteins were overabundant in CM-EVs from explants cultured alone (Exp) when comparing CM-EVs from Exp and explants co-cultured with embryos (Exp+Emb). (B) Twenty-one proteins were overabundant in CM-EVs from Exp when comparing CM-EVs from Exp and embryos cultured alone (Emb). (C) Seven proteins were overabundant in CM-EVs from Exp+Emb when comparing CM-EVs from Emb and Exp+Emb. Protein class, gene ontology, and pathways were identified using the PANTHER 18.0 Classification System (https://pantherdb.org/). Darker bars indicate the number of genes associated with each category name, while lighter bars represent the percentage of these genes relative to the total number of genes in that category.

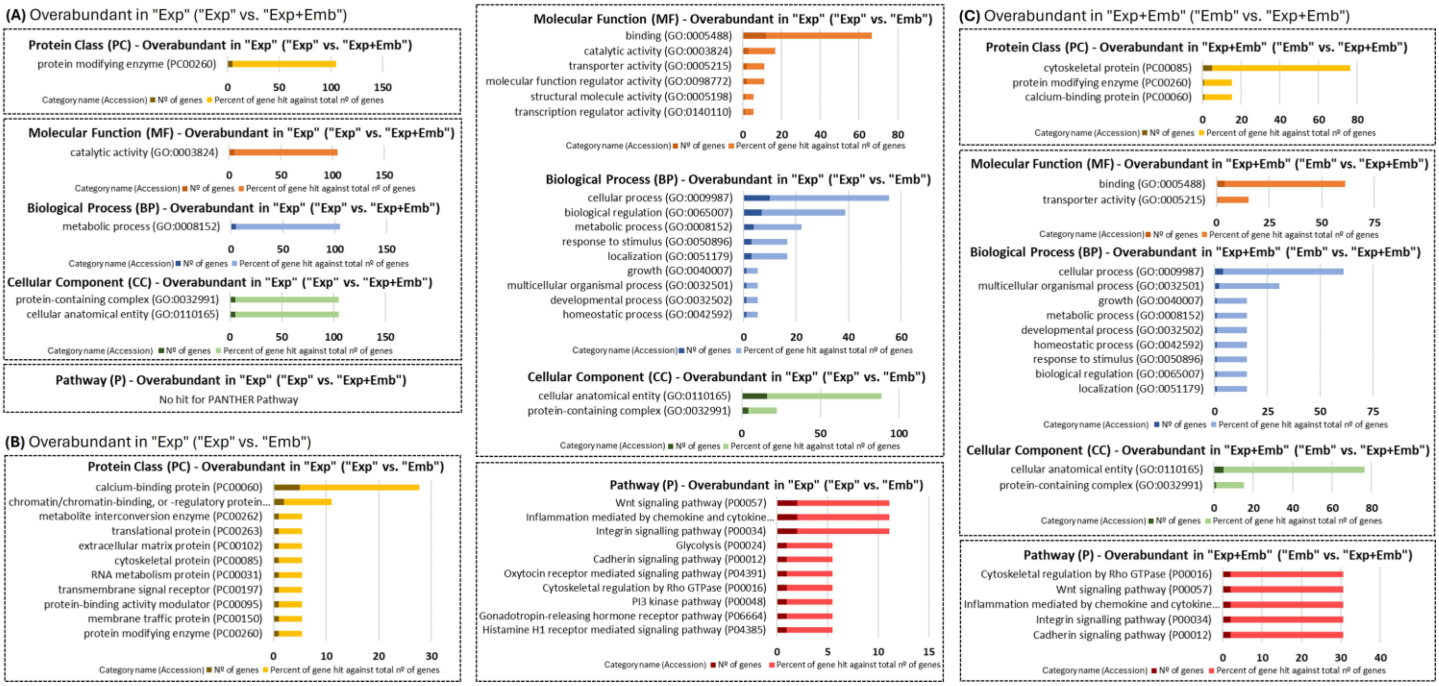

Figure S4. Functional Enrichment of Proteins Identified in Oviductal Fluid Extracellular Vesicles (OF-EVs) from Cyclic Heifers Compared to Conditioned Media Extracellular Vesicles (CM-EVs) from Explants Cultured Alone (Exp). (A) Fifty-five proteins exclusive to OF-EVs from Cyclic. (B) Fifty-six proteins exclusive to CM-EVs from Exp. (C) Two hundred ninety-three proteins are equally abundant among Exp *vs.* Cyclic. (D) One hundred fifty-five were differentially abundant among Exp *vs.* Cyclic. Protein class, gene ontology, and pathways were identified using the PANTHER 18.0 Classification System (https://pantherdb.org/). Darker bars indicate the number of genes associated with each category name, while lighter bars represent the percentage of these genes relative to the total number of genes in that category.

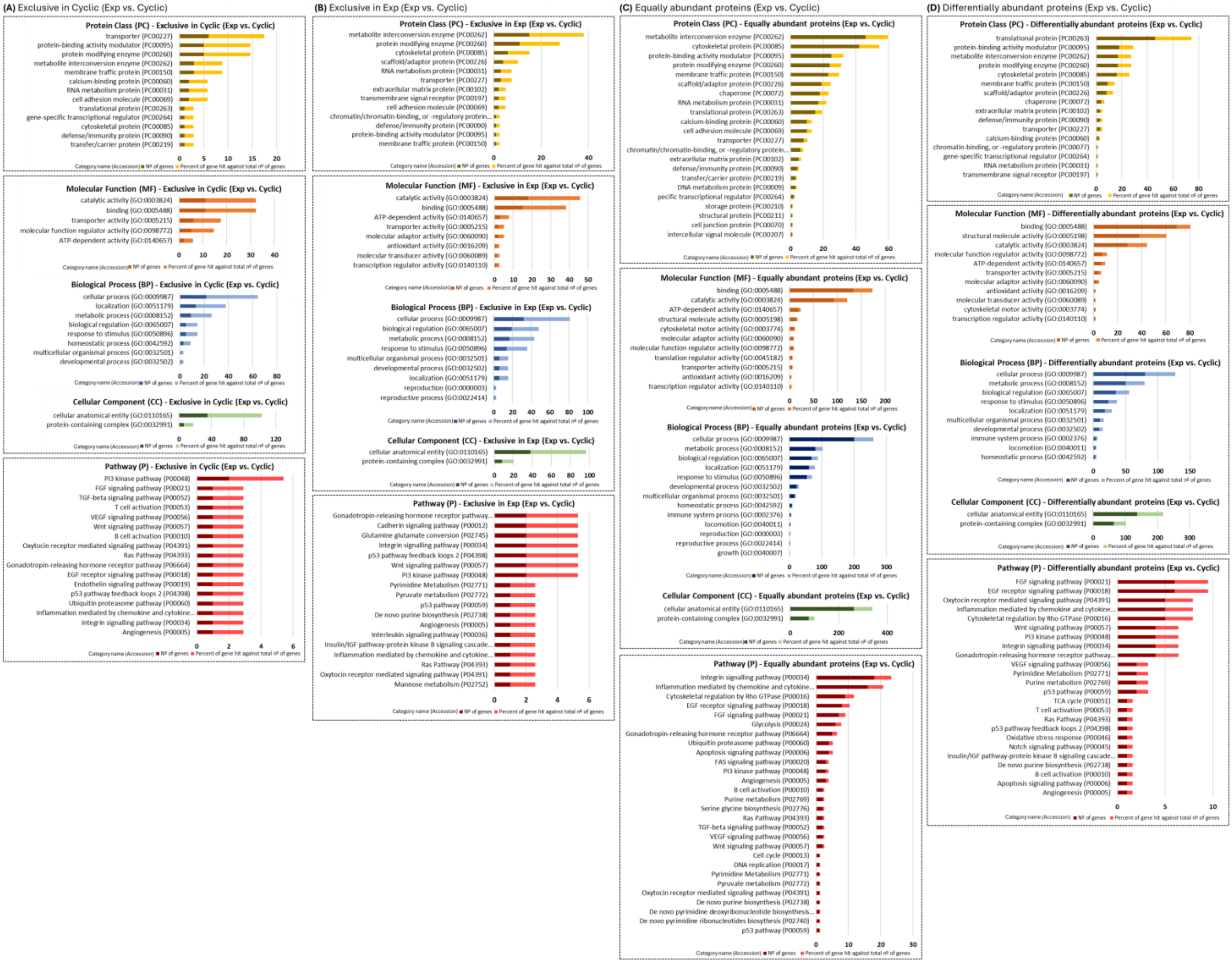

Figure S5. Functional Enrichment of Proteins Identified Oviductal Fluid Extracellular Vesicles (OF-EVs) from Pregnant Heifers Compared to Conditioned Media Extracellular Vesicles (CM-EVs) from Explants Cocultured with Embryos (Exp+Emb). (A) Forty-nine proteins exclusive to OF-EVs from Pregnant. (B) Fourteen proteins exclusive to CM-EVs from Exp+Emb. (C) Four hundred Twenty proteins were equally abundant among Exp+Emb *vs.* Pregnant. (D) One hundred sixteen were differentially abundant among Exp+Emb *vs.* Pregnant. Protein class, gene ontology, and pathways were identified using the PANTHER 18.0 Classification System (https://pantherdb.org/). Darker bars indicate the number of genes associated with each category name, while lighter bars represent the percentage of these genes relative to the total number of genes in that category.

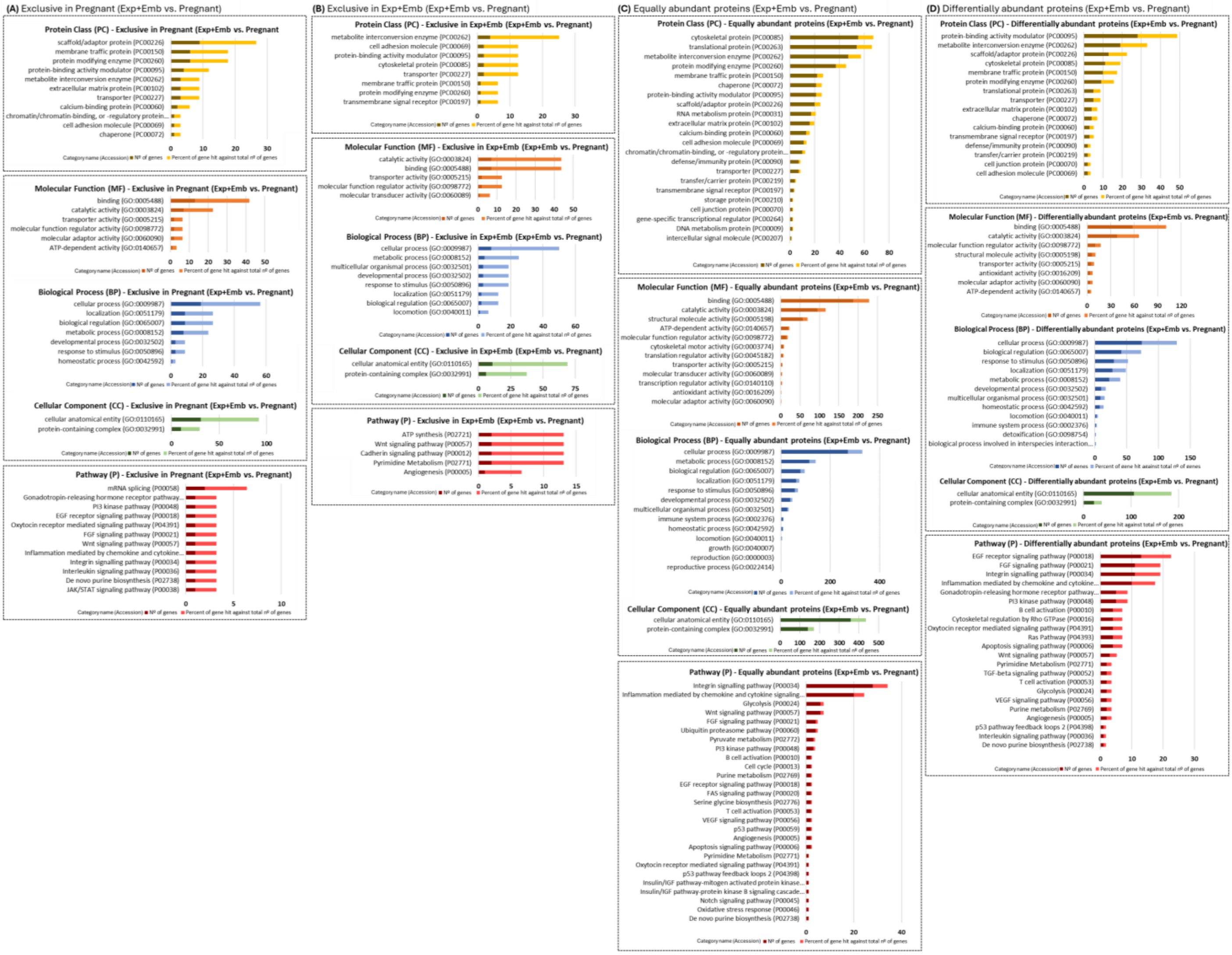

Supplement: Supplementary Figures [file mmc1.pdf]
